# Changes in gene expression in *Camelina sativa* roots and vegetative tissues in response to salinity stress

**DOI:** 10.1038/s41598-018-28204-4

**Published:** 2018-06-28

**Authors:** Zohreh Heydarian, Min Yu, Margaret Gruber, Cathy Coutu, Stephen J. Robinson, Dwayne D. Hegedus

**Affiliations:** 1 Agriculture and Agri-Food Canada, 107 Science Place, Saskatoon, SK S7N 0X2 Canada; 2 Department of Biotechnology, School of Agriculture, University of Shiraz, Bajgah, Shiraz, Fars Iran; 3 Department of Food and Bioproduct Sciences, University of Saskatchewan, Saskatoon, SK Canada

## Abstract

The response of *Camelina sativa* to salt stress was examined. Salt reduced shoot, but not root length. Root and shoot weight were affected by salt, as was photosynthetic capacity. Salt did not alter micro-element concentration in shoots, but increased macro-element (Ca and Mg) levels. Gene expression patterns in shoots indicated that salt stress may have led to shuttling of Na^+^ from the cytoplasm to the tonoplast and to an increase in K^+^ and Ca^+2^ import into the cytoplasm. In roots, gene expression patterns indicated that Na^+^ was exported from the cytoplasm by the SOS pathway and that K^+^ was imported in response to salt. Genes involved in chelation and storage were up-regulated in shoots, while metal detoxification appeared to involve various export mechanisms in roots. In shoots, genes involved in secondary metabolism leading to lignin, anthocyanin and wax production were up-regulated. Partial genome partitioning was observed in roots and shoots based on the expression of homeologous genes from the three *C. sativa* sub-genomes. Sub-genome I and II were involved in the response to salinity stress to about the same degree, while about 10% more differentially-expressed genes were associated with sub-genome III.

## Introduction

*Camelina sativa* (camelina, false flax or gold of pleasure) is a close relative of the model plant *Arabidopsis thaliana* (Arabidopsis) and the oilseed Brassica crops. Camelina was cultivated in Europe as an oilseed crop for food and fuel before being displaced by higher-yielding crops, such as oilseed rape/canola (*Brassica napus*) and wheat. Efforts to diversify annual crop rotation portfolios renewed interest in this ancient crop as it can be grown on marginal lands that are not well-suited for food crops and has the potential to be a low cost, high value oil and meal bio-feedstock^1–3^. It has enhanced drought, some degree of salinity and cold tolerance, displays early maturation, and requires fewer inputs compared to other oilseeds^4–6^. It is also naturally resistant to diseases that afflict canola, such as blackspot^7^, blackleg^8^, and stem rot^9^, as well as insect pests, such as the flea beetle and diamondback moth^10–12^.

Soil salinity presents a notable challenge to agriculture, which may be a consequence of human activities, such as irrigation, or alterations in rainfall patterns that reduce leaching of salts and minerals from soils. Lands that were once highly fertile have become less productive due to increased salt levels^13^. Furthermore, increasing pressure to use marginal lands for farming often means that growers struggle with naturally-occurring high levels of salt^14^. The effect of salt on plant growth and productivity is dependent on salt type, concentration, sensitivity of the crop, and the capacity of the plants to tolerate or mitigate the effects of salts alone or in combination^15^. Salinity inhibits plant growth and development by reducing water availability, similar to drought, and via ion toxicity^16^. Salt stress affects many cellular processes, including gene expression, protein synthesis, carbohydrate and lipid metabolism, osmotic and pH homeostasis^17^, and limits plant growth by impairing photosynthesis, metabolic processes and nutrient acquisition^18,19^.

While species and even genotypes may respond differently to stress, many stresses share the same set of general responses^20^. Since camelina is somewhat resistant to drought, some degree of salinity resistance is to be expected. Indeed, the ability to avoid drought by developing deep root systems or making metabolic adjustments^21^ is a common feature of both drought and salt tolerance. Typically, salt affects root elongation^22,23^ and root architecture by reducing cell size and cell division and altering differentiation patterns. For example, exposure to salt alters differentiation of the Casparian strip causing it to be unusually close to the root meristem^24^ which changes root architecture^25^ and the root gravitrophic response, halotropism^26^. In addition, cell cycle inhibition as a result of salt stress causes cells in the meristem to stop dividing; cells elongate at the root tip, but do not divide^27^ and root size is reduced^28^.

The *C. sativa* genome was suggested to have arisen from a genome triplication event^29^, a supposition supported by genome sequencing^30^. However, the evolutionary origin of the ancestral genomes and the polyploidization and post-polyploidization events that led to diploidization are not fully understood^30^. There is little evidence of fractionation bias in the *C. sativa* genome and the highly undifferentiated polyploid genome presents significant challenges for breeding and genetic manipulation^30–32^. Although tolerance to abiotic stresses is a well-known breeding target for many crops, the molecular and metabolic mechanisms underlying the responses to salt stresses are scarcely understood in well-studied crops, even less so for crops like camelina where genome information has only recently become available. Seed yield suffered substantially in camelina in response to moderate salt stress when examined over a complete life cycle^6^. Here we provide a detailed analysis of the gene expression patterns in roots and shoots of the type strain of *C. sativa*, the double haploid line DH55, in response to salt stress. Our objective was to determine the global transcriptome changes occurring in camelina as a function of salt stress at the point where plants began to switch to the reproductive phase.

## Material and Methods

### Plant Materials

*Camelina sativa* cv. DH55 seeds were sown in soil-less potting mixture^33^ in 10 × 10 × 8 cm square pots. All materials were available locally, except fritted trace elements (Frit Industries Inc., Ozark, USA). NaCl solutions at 192 and 213 mM were prepared to obtain solutions with electrical conductivities (EC) of 15 dSm^−1^ and EC 20 dSm^−1^ at 20 °C, respectively. 50 ml of tap water (EC 1,248 µSm-1) was applied daily to each pot and replaced with 50 ml of saline solution (EC 15 or EC 20 dSm-1) 19 days after sowing. The accumulation of salt in the pots was controlled by draining and the EC of the drained water was measured weekly. Plants were grown in a controlled growth chamber (16 h light/8 h dark, 20/17 °C) supplemented with halogen lights. When the plants entered the flowering stage after 20 days of initial salt stress, shoots and roots were collected from 10 replicates per treatment and their length, dry weight and fresh weight determined.

### Elemental and Chlorophyll Measurements

The quantity of elements and chlorophyll were determined in shoots of 33-day-old plants; this was 13 days after the initial salt treatment and before plants began to show severe symptoms of salinity stress. For element measurements, shoot samples were dried at 80 °C for 48 h and ground using liquid nitrogen. Samples were digested with nitric acid/hydrogen peroxide and examined for sulphur (S), copper (Cu), boron (B), calcium (Ca), iron (Fe), potassium (K), magnesium (Mg), manganese (Mn), sodium (Na), phosphorus (P), and zinc (Zn) content using a Perkin Elmer 5400 ICP analyzer by Agvise Laboratories Inc. (Benson, USA). Chlorophyll content was determined using a plant chlorophyll meter (Apogee Instruments Inc., Logan, UT, USA). Photosynthesis yield (FV/FM) was measured using a FV/FM meter (ADC Bioscientific Ltd, UK).

### RNA Sequencing and Data Analysis

Total RNA was extracted from root and shoot tissue (3 biological replicates) from 28-day-old plants (21 days after salt treatment began and before plants started to bolt) exposed to 15 dSm^−1^ NaCl and from non-stressed plants using the RNeasy Plant Mini Kit (Qiagen Inc.). cDNA libraries were prepared using the TruSeq Stranded mRNA and Total RNA Library Prep kits with TruSeq LT adaptors (Illumina Inc.). The tagged libraries were sequenced using a HiSeq. 2500 (Illumina Inc.).

The short-read sequence data from the 12 libraries were deposited in the NCBI GEO (GSE102422). Trimmomatic^34^ was used to discard low-quality reads, trim adaptor sequences, trim low quality nucleotides at the beginning or end of the read (PHRED33 quality score of less than 3), and discard short reads (under 21 nt). The retained high-quality reads were mapped to the *C. sativa* reference genome using STAR RNA-seq aligner program^35^ and transcripts available in the NCBI *C. sativa* release 100 (JFZQ00000000.1^30^). Expression levels for each gene were measured as counts^36,37^. For each gene, normalization for library size was performed by dividing the counts by the library size to yield CPM^38^. Further normalization was performed using DESeq to approximate a negative binomial distribution. The differential expression analysis of digital gene expression data software (edgeR) was used to calculate expression changes between libraries^36^. The Biological Coefficient of Variation (BCV) value was set to 2 according to the software’s instructions. Expression changes were said to be significant if the multiple test corrected p-value and the false discovery rate (FDR) were ≤ 0.05 and the absolute value of log 2 CPM was higher than 1. MAPMAN software was used to assign GO terms to unigenes based on molecular function, biological processes and cellular compartment^39^.

### Quantitative droplet digital PCR (ddPCR) analysis

Quantitative ddPCR was performed to compare the expression profiles of select genes as determined by RNA-Seq analysis. Total RNA was extracted from roots and shoots of 3 independent biological replicates (control and salt treatment) using the RNeasy Plant Mini kit (Qiagen) and cDNA synthesized using the SuperScript III First-Strand Synthesis kit (Thermo Fisher Scientific). 1 ng of cDNA was added to the PCR reaction mixture with 2 × Supermix (Bio-Rad), 10 µM of each primer and 3.25 nM of each probe in a final volume of 20 µl. The sequences of the primers and probes can be found in Supplemental Table 1. Data were analyzed using Quanta-Soft version 1.7.4.0917 (Bio-Rad) and the relative ratio of the candidate gene expression was calculated relative to the expression of *actin* reference gene by plotting the concentration of FAM over the HEX labelled probe according to the Bio-Rad ddPCR application guide.

### Statistical analysis

Plant growth measurements were expressed as the mean ± standard error for each treatment. Significant differences between treatments were determined by variance analysis (ANOVA) with a p-value of ≤0.05 and pair-wise comparisons were conducted using the Tukey’s Studentized Range (HSD) test using SAS Software 9.3 (TS1M2).

### Data availability statement

The RNA-Seq data were deposited to the NCBI Gene Expression Omnibus under the accession code GSE102422.

## Results

### Plant growth, photosynthetic capacity and elemental composition in response to salt treatment

**Figure 1:**
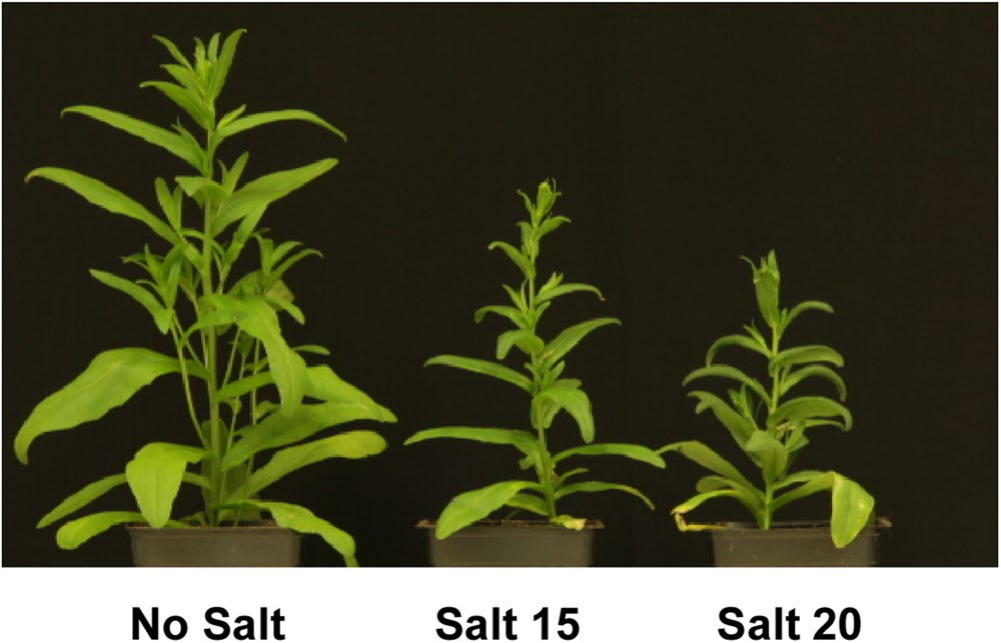
*C. sativa* DH55 grown in the absence or presence of salt (NaCl; 15 and 20 dSm^−1^). Photographs show plants 28 days after sowing with salt being applied 20 days after sowing.

**Figure 2:**
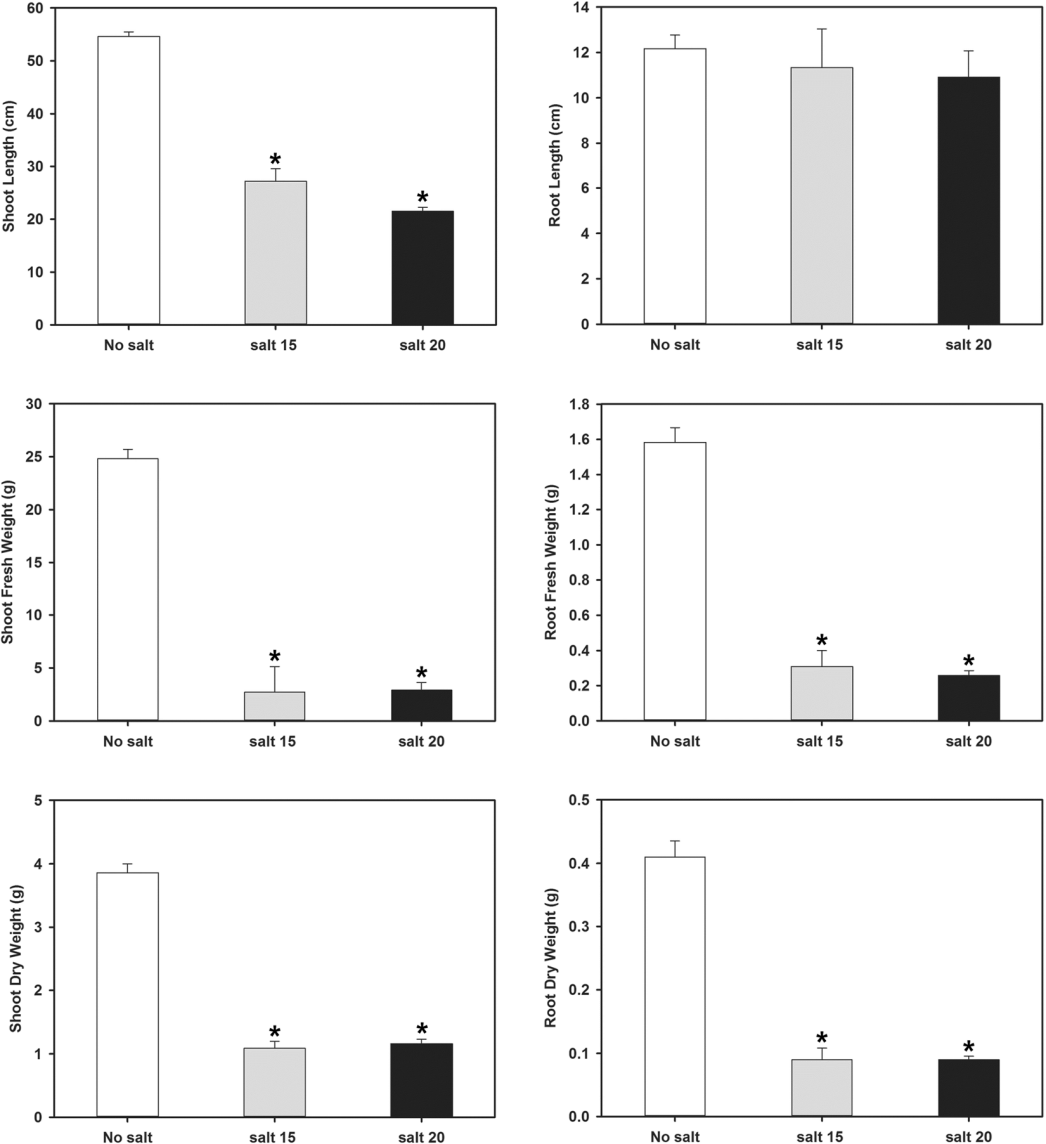
Root and shoot development of *C. sativa* DH55 grown in the absence or presence of salt (NaCl; 15 and 20 dSm^−1^). Panels show shoot and root length, shoot and root fresh weight, and shoot and root dry weight 33 days after sowing with salt being applied 20 days after sowing. Error bars indicate standard error (n = 10). A two-way ANOVA and Tukey’s post-test were used to detect significant differences between groups. Asterisks (*) above bars indicate values that are significantly different (p < 0.05) from the control (no salt).

**Figure 3:**
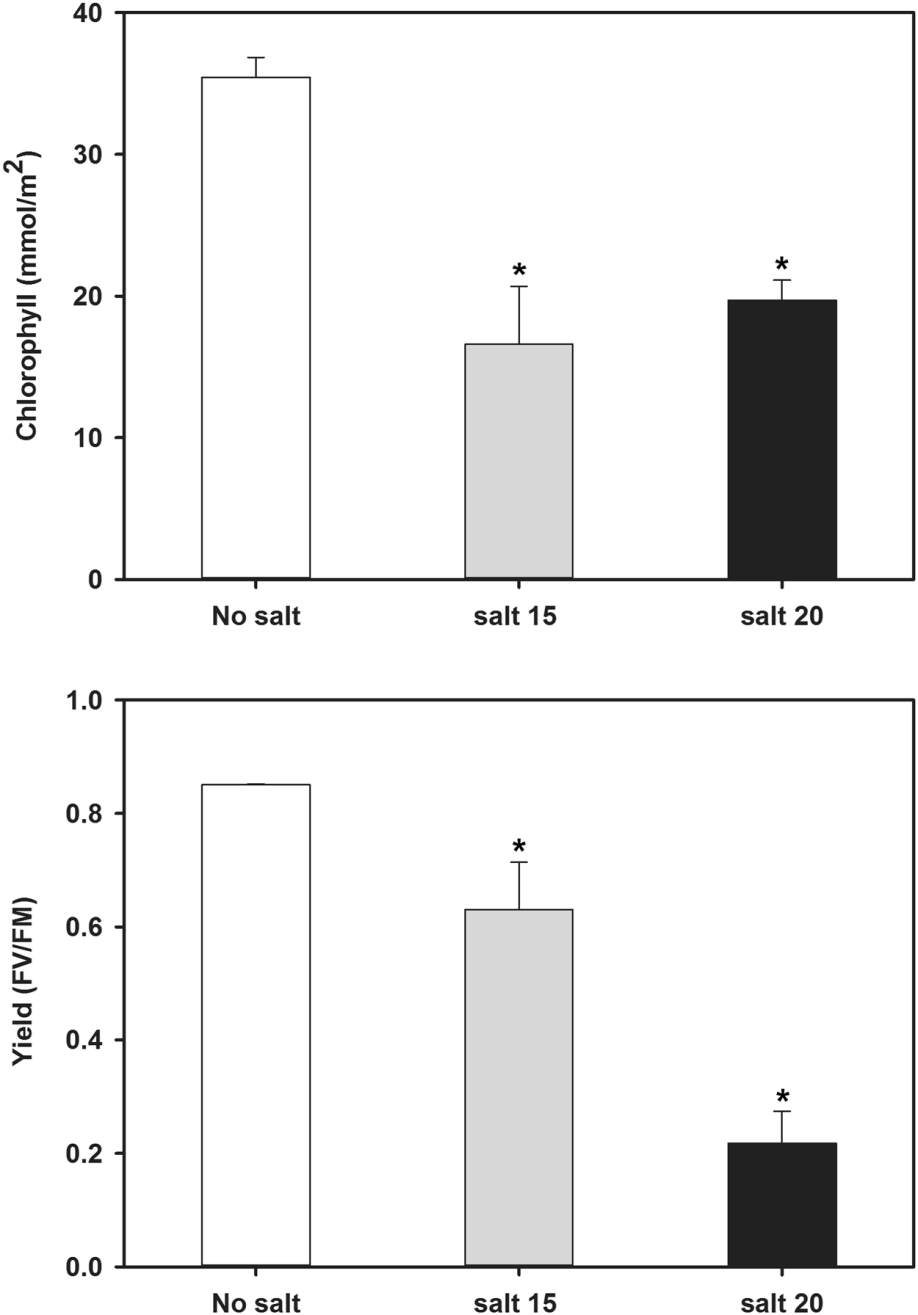
Photosynthetic capacity of *C. sativa* DH55 grown in the absence or presence of salt (NaCl; 15 and 20 dSm^−1^). Panels show chlorophyll content (A) and chlorophyll fluorescence yield (B) 33 days after sowing with salt being applied 20 days after sowing. Error bars indicate standard error for chlorophyll concentration (n = 12) and for chlorophyll yield (n = 6). A two-way ANOVA and Tukey’s post-test were used to detect significant differences between groups. Asterisks (*) above bars indicate values that are significantly different (p < 0.05) from the control (no salt).

**Figure 4:**
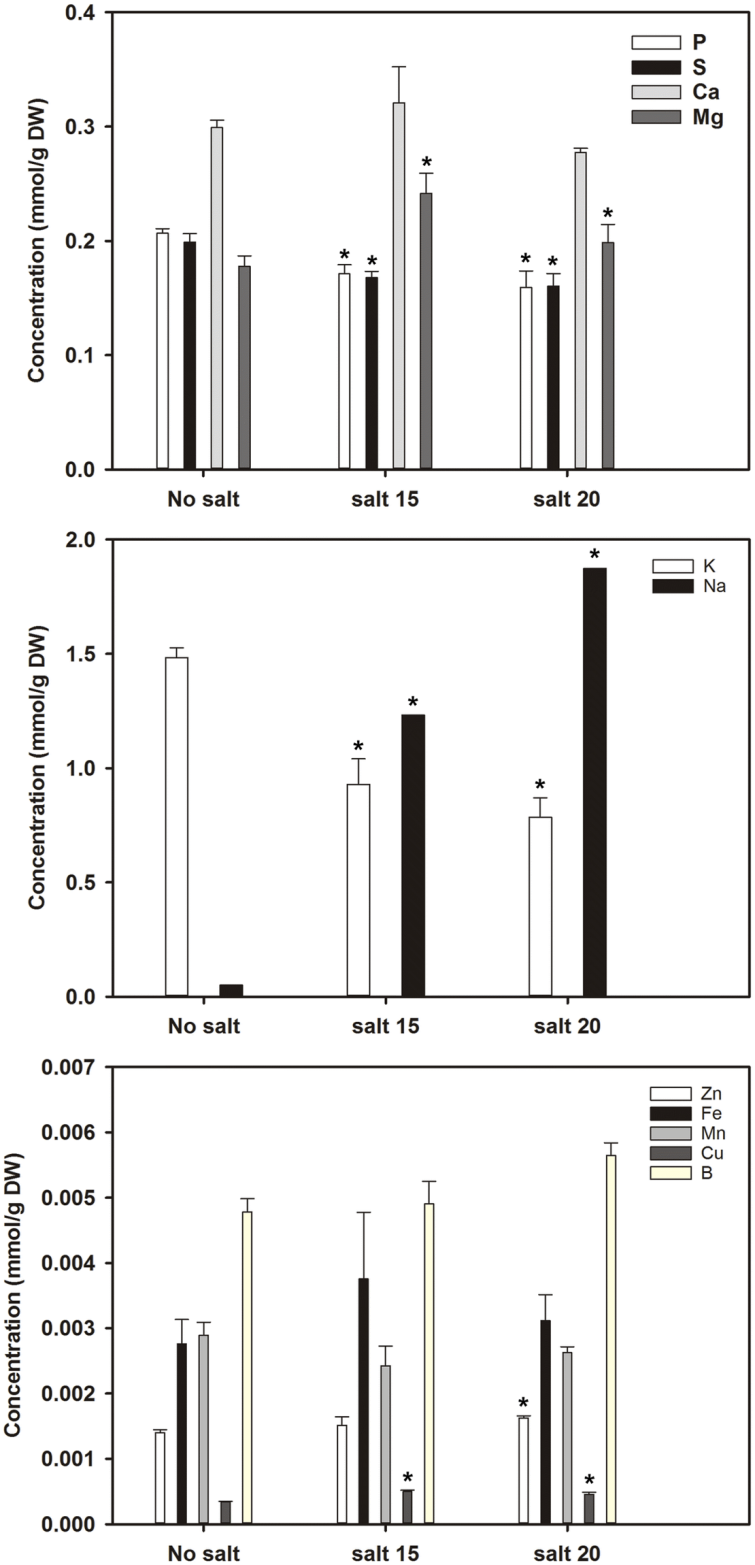
Element content in shoots of *C. sativa* DH55 grown in the absence or presence of salt (NaCl; 15 and 20 dSm^−1^). Panels show macro-element (P, S, Ca, Mg, Na and K) and micro-element (Zn, Fe, Mn, Cu, and B) content in shoots of plants 33 days after sowing with salt being applied 20 days after sowing. Error bars indicate standard error (n = 3). A two-way ANOVA and Tukey’s post-test were used to detect significant differences between groups. Asterisks (*) above bars indicate values that are significantly different (p < 0.05) from the control (no salt).

In previous studies, camelina salt tolerance was examined using a salt mixture that simulated naturally saline soils found on the Canadian/North American prairies^3,6^. The current study used NaCl at the same EC values as this provided for better comparison to most other published studies on salinity tolerance which use only NaCl. Salt concentration impacted the length, fresh and dry weights of roots and shoots (Figs 1, 2). Shoot length was significantly reduced at both salt concentrations tested; however, root length was not significantly affected resulting in a higher root/shoot length ratio during salt treatment. The fresh and dry weights of both roots and shoots were severely reduced at both salt concentrations tested and to approximately the same degree. Chlorophyll content and photosynthetic yield decreased significantly as the concentration of salt increased (Fig. 3). Twelve days after salt treatment, the content of P, K, and S decreased significantly in shoots, while Cu, Mg and Na content increased at both the 15 and 20 dSm-1 salt levels. Zn content increased in shoots only at the 20 dSm^−1^ salt level, while the contents of Ca, Fe, B and Mn did not change significantly (Fig. 4).

### Global changes in the camelina transcriptome in response to salt treatment

**Figure 5:**
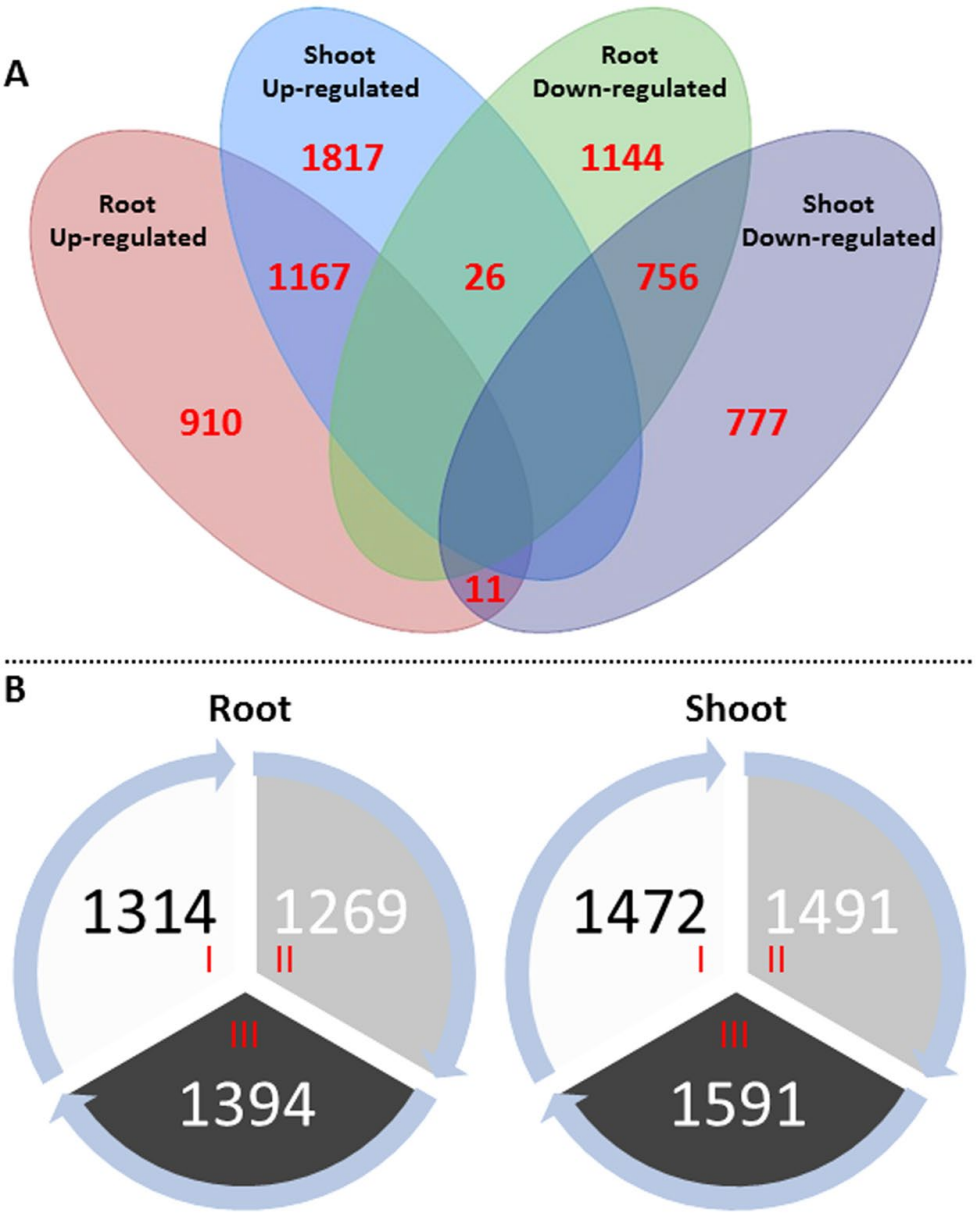
The number of genes that were differentially-expressed in *C. sativa* DH55 in the absence or presence of salt (NaCl; 15 dSm^−1^). Panel A: Venn diagram showing the number of differentially-expressed genes in shoots and roots of 4-week-old plants after treatment with salt for 3 weeks. Panel B: Diagram showing the number of differentially-expressed genes in shoots and roots from each of the 3 *C. sativa* sub-genomes (I, II and III).

To further explore the response to salinity stress in camelina, the transcriptomes in roots and shoots of plants grown in normal soil and soil treated with salt (15 dSm^−1^) were compared. In total, 2080 genes were up-regulated and 1924 genes were down-regulated in salt-treated roots (FDR ≤ 0.05 and expression < 2 fold). Of these, 1314 genes were assigned to *C. sativa* sub-genome I, 1269 genes to sub-genome II, 1394 genes to sub-genome III (Fig. 5). In shoots, 3058 genes were up-regulated and 1543 genes were down-regulated in response to salt treatment (FDR ≤ 0.05 and expression < 2 fold). Of these, 1472 genes could be assigned to *C. sativa* sub-genome I, 1491 genes to sub-genome II, 1591 genes to sub-genome III. Among them, 910 genes were up-regulated and 1144 genes were down-regulated only in roots, while 1817 genes were up-regulated and 777 genes down-regulated only in shoots. In total, 1960 differentially-expressed genes were common in shoots and roots, of which 1167 genes were up-regulated and 756 were down-regulated. Only 26 genes were up-regulated in shoots, but down-regulated in roots, whereas only 11 genes were down-regulated in shoots, but up-regulated in roots. To validate the RNA-Seq results, a select group of differentially-expressed genes (both up- and down-regulated) were selected for quantitative ddPCR analysis (Supplemental Fig. 1). The expression profiles of the genes as examined by both methods were highly similar. A complete annotation of the genes that were differentially-expressed in roots and shoots of *C. sativa* DH55 can be found in Supplemental Data and are discussed in more detail below. Gene expression heat maps superimposed on MAPMAN GO pathways are provided in Supplemental Fig. 2.

### Overview of systems and biochemical pathways affected by salt treatment

#### Photosynthesis and photorespiration

Salt treatment negatively impacted photosynthetic ability in camelina (Fig. 3). In total, 22 genes related to photosynthesis were down-regulated in salt-treated shoots; 6 of these were related to photosystem II (PSII), 2 were related to PSI, 2 were related to the electron transport chain and 11 were related to the Calvin cycle (Supplemental Data). In contrast, genes involved in photorespiration, such as those encoding glycolate oxidase (Csa11g030110, Csa06g046560, and Csa10g026850), were up-regulated in shoots. Interestingly, the expression of photosynthesis-related genes was altered in roots in response to salt treatment, including 6 genes related to PSII and 1 gene related to PSI which were down-regulated. Genes encoding ribulose bisphosphate carboxylase small chain 1 A (Csa16g026850, Csa05g079910 and Csa07g032360), an enzyme involved in both photosynthesis (Calvin Cycle) and photorespiration, were up-regulated in roots. A gene encoding a chlorophyll-binding protein involved in PSII (Csa06g008820) was up-regulated around 200-fold.

#### Carbohydrate metabolism and respiration

An adaptive response to salinity stress accrues from shifts in carbohydrate metabolism and partitioning^40^. This reduces growth rate; however, the accumulated soluble sugars function as osmolytes to maintain turgor and to protect cell membranes and proteins from damage^41^. Genes encoding enzymes involved in starch catabolism, such as α-amylase (Csa15g030630, Csa19g028300, and Csa01g023510) were down-regulated in shoots, while those encoding β-amylase (Csa01g030170, Csa19g036120, and Csa15g048800) were up-regulated. Two genes encoding α-amylases (Csa10g017970 and Csa12g029250) and three genes encoding sucrose synthase 3 (Csa02g005510, Csa08g052190, and Csa13g054950) were up-regulated in shoots and roots (Supplemental Data).

Increasing soluble sugars is a common aspect of most abiotic stress responses as they can be used as energy sources, as osmolytes or as signaling molecules^20,41^. Genes encoding enzymes involved in the synthesis or metabolism of soluble sugars, such as melibiose, myo-inositol-1-phosphate, raffinose, and trehalose were up-regulated in salt-treated shoots. The most affected genes were Csa05g063400 (galactinol synthase 2) which was up-regulated 54-fold and Csa10g047220, Csa11g057610, Csa12g079610 (raffinose synthase) which were up-regulated 3 to 19-fold. A different set of genes involved in simple sugar production was up-regulated in roots, including Csa00532s180, Csa06g021410 and Csa19g003320 (galactose mutarotase), Csa07g061680 and Csa16g052330 (galactinol synthase 4), and Csa17g030990, Csa14g030990 and Csa03g027670 (trehalose-phosphatase/synthase 9). Several genes that were up-regulated in roots and shoots encoded myo-inositol oxygenases.

Genes related to glycolysis and fermentation were mostly up-regulated in shoots and roots, while most genes related to the glyoxylate and Krebs cycles were down-regulated in shoots. Interestingly, genes encoding citrate synthase 3 (Csa04g059380, Csa05g008770 and Csa06g048940) and citrate synthase 5 (Csa16g005860) were the only Krebs cycle genes that were up-regulated in shoots and/or roots. The expression of genes involved in mitochondrial function was highly affected after salt treatment. Genes encoding several types of alternative oxidases were highly up-regulated, including those encoding alternative oxidase 1D (Csa17g051700, Csa14g042300 and Csa03g036700) which were up-regulated 134 to 240-fold in shoots and 16 to 42-fold in roots, Csa02g072900 (alternative oxidase 2) which was up-regulated 52-fold in shoots and 80-fold in roots, and alternative oxidase 1 A (Csa01g025600, Csa15g036960 and Csa19g031590) which were up-regulated 6 to 32-fold in shoots and 14 to 22-fold in roots. Csa15g035960 (alternative oxidase 1B) was highly up-regulated (784-fold), but only in shoots.

#### Macromolecule synthesis related to nitrogen and lipid metabolism

**Figure 6:**
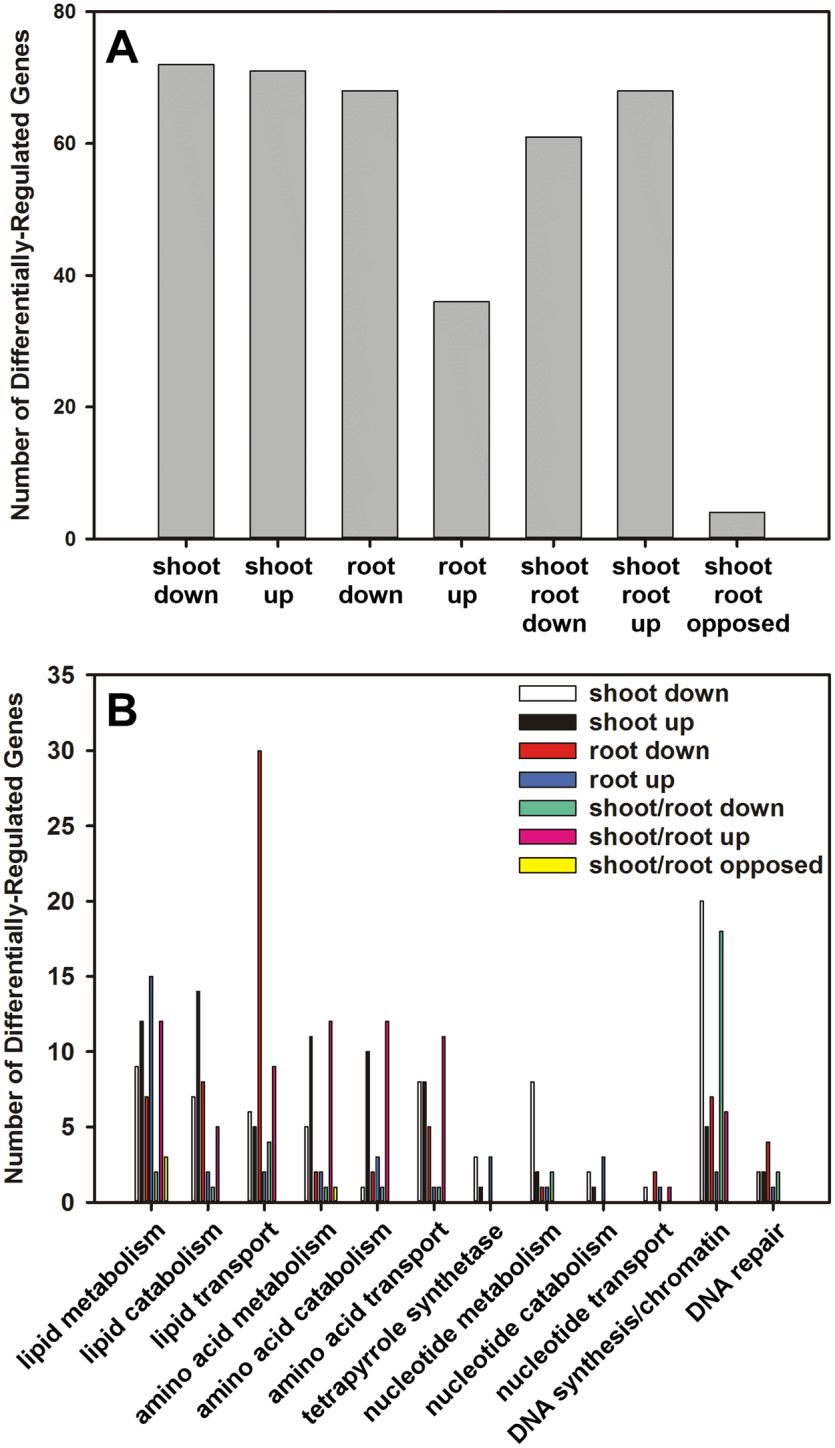
Gene ontology (GO) overview of genes involved in macromolecule synthesis that were differentially-expressed in *C. sativa* DH55 in response to salt treatment. Panels show a general overview of differentially-expressed genes (**A**) and a breakdown according to lipid, amino acid, and nucleotide metabolism (**B**). Genes with an FDR and P value ≤ 0.05 and an absolute value of log 2 FC higher than 1 were considered significant.

Twice as many genes related to macromolecule biosynthesis were down-regulated than were up- regulated in roots, while only a few genes were regulated oppositely in roots and shoots (Fig. 6A). Many genes involved in lipid, amino acid, and nucleotide metabolism were differentially regulated in response to salt stress and their expression profiles were comparable in shoots and roots.

Increased nitrogen metabolism is observed in halotolerant plants, such as *Salicornia europaea*^42^, and in the roots of salt-resistant rice cultivars^43^. Furthermore, over-expression of glutamine synthetase enhances salt tolerance in Arabidopsis^44^. In contrast, camelina genes encoding glutamine synthetase 1 (Csa20g024450) and glutamate dehydrogenase 3 (Csa19g006110 and Csa15g003740) were down-regulated in shoots. Genes encoding nitrate reductase 2 (Csa03g040490) were down-regulated in roots, while those encoding glutamine synthase R1 (Csa11g051370) were down-regulated in shoots and roots (Supplemental Data).

Plants subjected to salt stress accumulate different amino acids which variably act as osmolytes, regulate ion transport, control stomatal departure, or detoxify heavy metals. Furthermore, some amino acids affect the synthesis and activity of different enzymes, and regulate plant redox-homeostasis^45^. Five genes related to amino acid synthesis were down-regulated in shoots, including Csa20g023980 (valine tolerance 1, a regulatory subunit of acetohydroxy acid synthase and the first committed enzyme in the branched chain amino acid biosynthesis pathway) and Csa01g003320 (cysteine synthase 26). Fifteen genes were up-regulated in shoots, including Csa04g006420, Csa06g002010 and Csa09g004390 (glutamate decarboxylase 4), Csa15g024000 (glutamate decarboxylase 5), and Csa06g020990 (glutamine-dependent asparagine synthase 1) (Fig. 6B; Supplemental Data).

Nine genes related to lipid metabolism were down-regulated in shoots and 12 genes were up-regulated, while 15 genes were up-regulated and 8 genes down-regulated in roots (Fig. 6B). Among them, those encoding 3-ketoacyl-CoA synthase 2, 12 and 19 involved in fatty acid synthesis were down-regulated in shoots, while genes involved in lipid degradation, such as those encoding lipases (Csa13g028770, Csa20g039550 and Csa08g018560), were down-regulated. Many genes related to lipid metabolism and catabolism were also up-regulated in shoots, for example, Csa09g035780 (lecithin:cholesterol acyltransferase) was up-regulated 70-fold. Genes related to lipid catabolism were highly regulated in roots and included Csa08g018530, Csa20g039470 and Csa13g028700 (lipase class 3) and Csa20g036070 (fatty acid reductase 7) (Supplemental Data).

#### Growth and development

**Figure 7:**
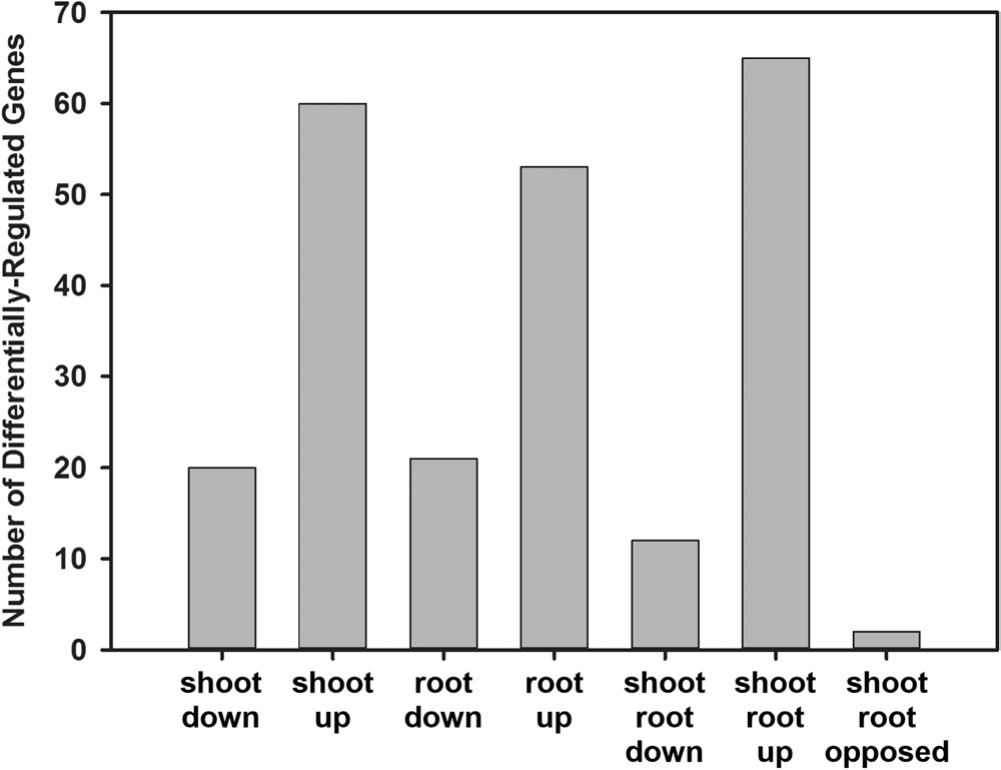
Gene ontology (GO) overview of genes involved in development that were differentially-expressed in *C. sativa* DH55 in response to salt treatment. Genes with an FDR and P value ≤ 0.05 and an absolute value of log 2 FC higher than 1 were considered significant.

In response to salt stress, three times more genes involved in some aspect of plant development were up-regulated than down-regulated (Fig. 7); many encoded late embryogenesis abundant proteins (LEA) which were up-regulated as much as 553-fold in roots and 173-fold in shoots (Supplemental Data). Genes encoding Responsive to Desiccation (RD26) (Csa10g015440, Csa11g016750, and Csa12g023460), Senescence-Associated Protein 29 (SAG29) (Csa20g018520 and Csa13g015780) and Dormancy-Associated Protein-like 1 (DYL1) (Csa14g035950, Csa17g039180 and Csa03g031630) were up-regulated in shoots and roots. Several genes encoding NAC transcription factors, namely NAC1, NAC2, NAC13, NAC32, NAC89, NAC102 and NAC109, were also up-regulated. Csa12g029240 encodes a nodulin MtN3 family protein and was highly up-regulated in roots (50-fold), but down-regulated in shoots (15-fold).

The expression of cell wall composition-related genes was affected to a much greater degree in roots than in shoots during salt treatment. It should be noted that camelina root length was not affected by salt stress (Fig. 2), though root mass was. In total, 17 genes were down-regulated and 14 genes were specifically up-regulated in shoots; however, 256 genes were down-regulated and 7 genes were specifically up-regulated in roots (Supplemental Data). Genes involved in primary cell wall modification and growth were highly affected in shoots, including those encoding expansins A4, 5, 8 and B3 which were down-regulated in shoots; however, genes encoding expansin A10 (Csa03g028750, Csa17g034120 and Csa14g031940) were up-regulated. While some genes encoding pectin lyases were up-regulated (Csa01g009040 and Csa15g009700), others were down-regulated (Csa07g059670 and Csa18g036730). In roots, several genes involved in pectin synthesis or modification were up-regulated, including those encoding FASCICLIN-like arabinogalactan protein 5 precursor and arabinogalactan protein 14 (5-fold). Conversely, genes encoding arabinogalactan proteins 1, 7, 13, 16, 22, and 24 and those encoding expansins 12, A1, A14, A15, and B1 were down-regulated. In addition, genes encoding proteins involved in pectin catabolism, such as root hair-specific protein 8, 12 and 14 were down-regulated in roots. Interestingly, Csa07g052560 (arabinogalactan protein 2; AGP2) and Csa06g054090 (pectin methyl-esterase inhibitor; PMEI) were down-regulated in roots, but up-regulated in shoots.

#### Growth regulators

Auxin: The reduction in plant growth and development under stress conditions may be an outcome of altered auxin accumulation and/or redistribution^46^. In camelina, the expression of genes encoding auxin-responsive proteins or enzymes involved in auxin synthesis was altered in both roots and shoots in response to salt treatment (Supplemental Data). Two genes encoding a cytochrome P450 involved in tryptophan metabolism (Csa11g003080 and Csa10g002730) were up-regulated in shoots. These enzymes convert trytophan to indo-3-acetaldoxime (IAOx), a precursor for indole acetic acid (IAA) and indole glucosinolates. Csa07g040770 encodes a PIN1 protein involved in the maintenance of auxin gradients and was down-regulated in shoots. Csa11g090640 encodes Ethylene Insensitive Root 1 (EIN1), a root-specific auxin transporter, and was down-regulated in roots, as were Csa17g094210 and Csa14g064340 that encode WaG1 proteins involved in auxin polar transport and root development. Genes encoding dormancy/auxin associated family proteins (Csa16g007290 and Csa05g021450) and genes involved in IAA biosynthesis (Csa03g046940, Csa14g049310 and Csa17g070920) were up-regulated in shoots and roots.

Gibberellin (GA): GA has a negative effect on salt tolerance^47^. Indeed, most of the genes involved in GA synthesis and signaling were down-regulated in shoots in response to salt treatment (Supplemental Data), including those encoding a negative regulator of GA responses, RGA-like 1 (Csa16g023800, Csa07g030410 and Csa05g075230) and gibberellin 20-oxidase 3 (Csa20g009560). Csa03g002450, Csa14g002470 and Csa17g001180 encode gibberellin 2-oxidase 6 involved in changing GA34 and GA8 to GA4 and GA1 (the active form of GA) and were the only GA biosynthetic genes to be up-regulated in shoots, while genes encoding gibberellin 3-oxidase 1 (Csa17g021160, Csa14g018700 and Csa03g019370) and gibberellin 3-oxidase 2 (Csa07g059520) were up-regulated in roots.

Brassinosteroid (BR): BR has been demonstrated to have a positive role in salt and drought stress^48^. Interestingly, genes involved in BR synthesis (Csa15g017890 and Csa01g016080) were down-regulated in shoots, while genes encoding brassinosteroid-6-oxidase 1 (Csa12g082470 and Csa10g045200) and sterol methyltransferase 3 (Csa16g040220, Csa09g080790 and Csa07g047620) were down-regulated in roots in response to salt treatment in camelina (Supplemental Data). The other homeologue encoding brassinosteroid-6-oxidase 1 (Csa11g05505) was down-regulated in both roots and shoots, as were genes encoding BR Suppressor 1 (BRS1) (Csa10g011910 and Csa12g016550). Conversely, Csa04g009350, encoding a sulpho-transferase 12 involved in BR synthesis, was highly up-regulated in roots (18-fold) and shoots (608-fold).

Cytokinin: Cytokinins are involved in salinity and high temperature tolerance in plants^49^. In camelina, two genes (Csa16g001090 and Csa07g001160) encoding cytokinin oxidase/dehydrogenase 6 involved in degradation of cytokinins were down-regulated in shoots, but a gene encoding cytokinin oxidase 3 (Csa02g061320) was up-regulated (Supplemental Data). The gene encoding cis-zeatin O-beta-D-glucosyltransferase (Csa14g027610) was also up-regulated in shoots. No cytokinin-related genes were differentially-regulated in roots in response to salt treatment.

Ethylene: Ethylene has a profound effect on salinity stress as low levels induce mechanisms required for stress tolerance; however, high levels negatively impact plant growth^50,51^. Seventeen genes involved in ethylene biosynthesis or signaling were up-regulated in shoots (Supplemental Data). Among them were genes encoding the Ethylene Response Factor domain proteins ERF11 (Csa14g036010, Csa17g039230 and Csa03g031680), ERF1 (Csa01g027770), ERF5 (Csa18g002580 and Csa20g081690), as well as ACC oxidase 2 (Csa09g097000), ACC synthase 2 (Csa14g001660) and ACC synthase 7 (Csa10g016710 and Csa12g025860).

In roots, eight genes related to ethylene synthesis or signalling were down-regulated, including those encoding Ethylene and Salt induced 3, ESE3 (Csa13g031030, Csa08g022870 and Csa20g047450) and ERF105 (Csa11g082690 and Csa18g022000). Genes encoding ethylene-forming enzyme (Csa14g006260 and Csa17g008270) and ACC synthase 8 (Csa10g003330) were up-regulated in roots. Interestingly, a gene encoding another ERF1 homeologue (Csa19g033660) was up-regulated in roots and in shoots, as were the two other genes encoding ACC synthase 2 (Csa17g001950 and Csa03g001750) and another gene encoding ACC synthase 7 (Csa11g018190). In contrast, genes encoding ERF106 (Csa08g056810, Csa20g010010 and Csa13g009640) and a gene encoding ACC oxidase 1 (Csa00441s160) were down-regulated in roots and shoots.

Jasmonic acid (JA): A highly positive correlation exists between JA concentration and salt tolerance in plants^46^. Indeed, several genes encoding enzymes involved in JA biosynthesis were up-regulated in shoots and/or roots (Supplemental Data), including allene oxide cyclase 2 (Csa09g008870), lipoxygenase 4 (Csa09g076110, Csa07g039620 and Csa16g035210), and lipoxygenase 3 (Csa03g021200). Interestingly, another homeologue of Csa09g008870 (Csa06g005340) was down-regulated in roots, but up-regulated in shoots.

Abscisic acid (ABA): Increase in ABA under lower water availability helps plants acclimate by closing stomata and promoting the accumulation of osmo-protectants^21^. Genes related to the ABA pathway were up-regulated in shoots (Supplemental Data); two encoded GEM-Related 5 (Csa13g015810 and Csa20g018560) which is required for the maintenance of dormancy by ABA and another aldehyde oxidase 2 (Csa04g022460) which functions in ABA biosynthesis. In roots, genes encoding ABA responsive element-binding Factor 1 (ABF1) (Csa17g085550), ABF2 (Csa14g049830), ABF4 (Csa15g027010) and 9-cis-epoxy carotenoid dioxygenase 2 (NCED2) (Csa11g030120, Csa06g046570 and Csa10g026860), a key enzyme in ABA biosynthesis, were up-regulated. Other positive regulators of ABA signalling were up-regulated in both shoots and roots, including ABF2 (Csa17g071450), ABF3 (Csa11g008990, Csa12g009690 and Csa10g008210), ABF4 (Csa01g022300), NCED3 (Csa15g018820, Csa01g017000 and Csa19g021150) and HVA22 D (Csa10g018030 and Csa11g019700). Interestingly, genes encoding negative regulators were also up-regulated in roots and/or shoots, for example, ABA deficient 1 (Csa11g103900, Csa02g075650 and Csa18g040510) and the ABA-induced protein phosphatase 2 C (HA1) (Csa14g008830, Csa17g010800 and Csa03g010830).

#### Secondary metabolism

**Figure 8:**
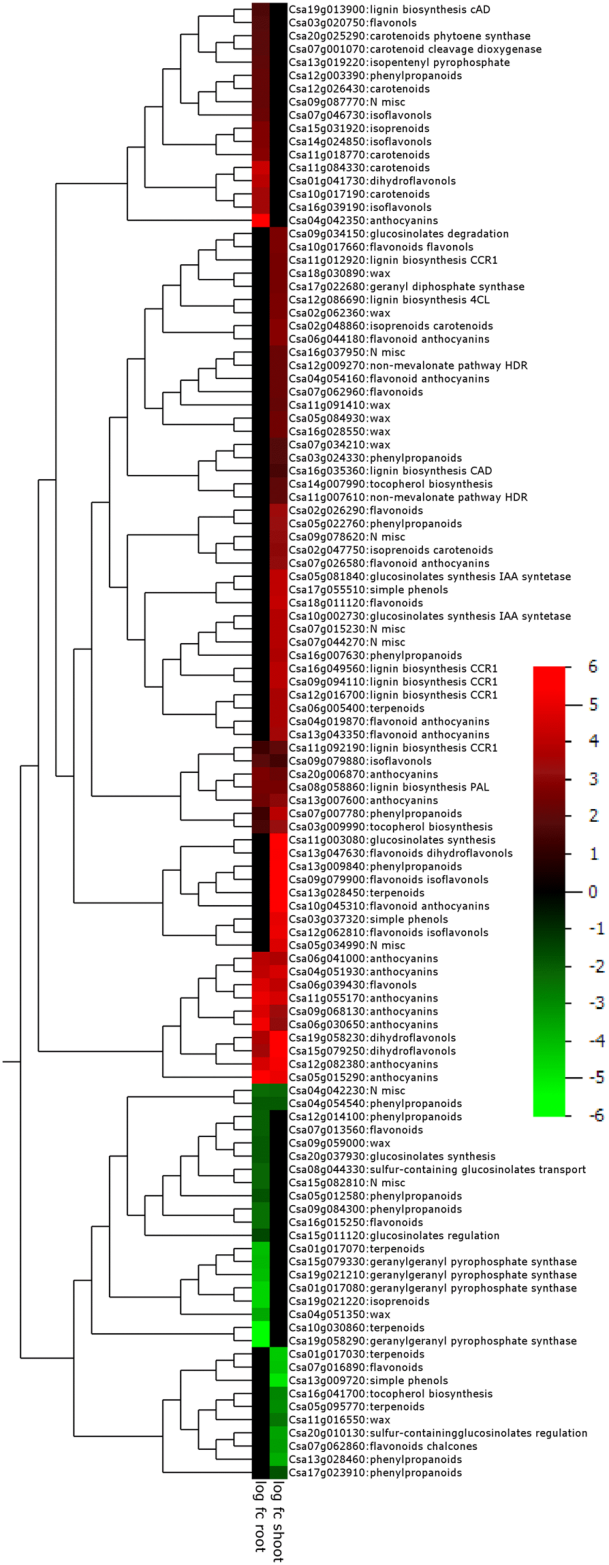
Hierarchical clustering analysis of genes involved in secondary metabolism that were differentially-expressed in *C. sativa* DH55 in response to salt treatment. Redder color indicates that more transcript accumulated and greener indicates less, while black indicates that gene expression was not significantly affected. Genes with an FDR and P value ≤ 0.05 and an absolute value of log 2 FC higher than 1 were considered significant.

The number of differently-regulated genes related to secondary metabolism was 3 times higher in shoots than in roots (Fig. 8). Genes involved in terpene synthesis (synthase and cyclase), wax metabolism, glucosinolate synthesis, catabolism and degradation, lignin biosynthesis, alkaloid metabolism, anthocyanin production, flavonoid and isoflavonol metabolism were up-regulated in shoots (Supplemental Data). Specifically, genes involved in anthocyanin (Csa04g042350) and carotenoid (Csa04g042350) pathways were highly up-regulated (76 and 20-fold, respectively). Down-regulated genes in shoots included those encoding terpene synthase 14 (Csa07g062860), and terpenoid cyclase (Csa01g017030), as well as MYB29 (Csa13g009720) and MYB79 (Csa20g010130) which are involved in glucosinolate biosynthesis^52^. Secondary metabolism genes that were down-regulated in roots included Csa10g030860 (baruol synthase 1 involved in triterpene biosynthesis) and Csa19g021220 (terpene synthase 19). Interestingly, only one gene involved in flavonoid and anthocyanin biosynthesis was commonly up-regulated in shoots and roots.

#### Stress response

**Figure 9:**
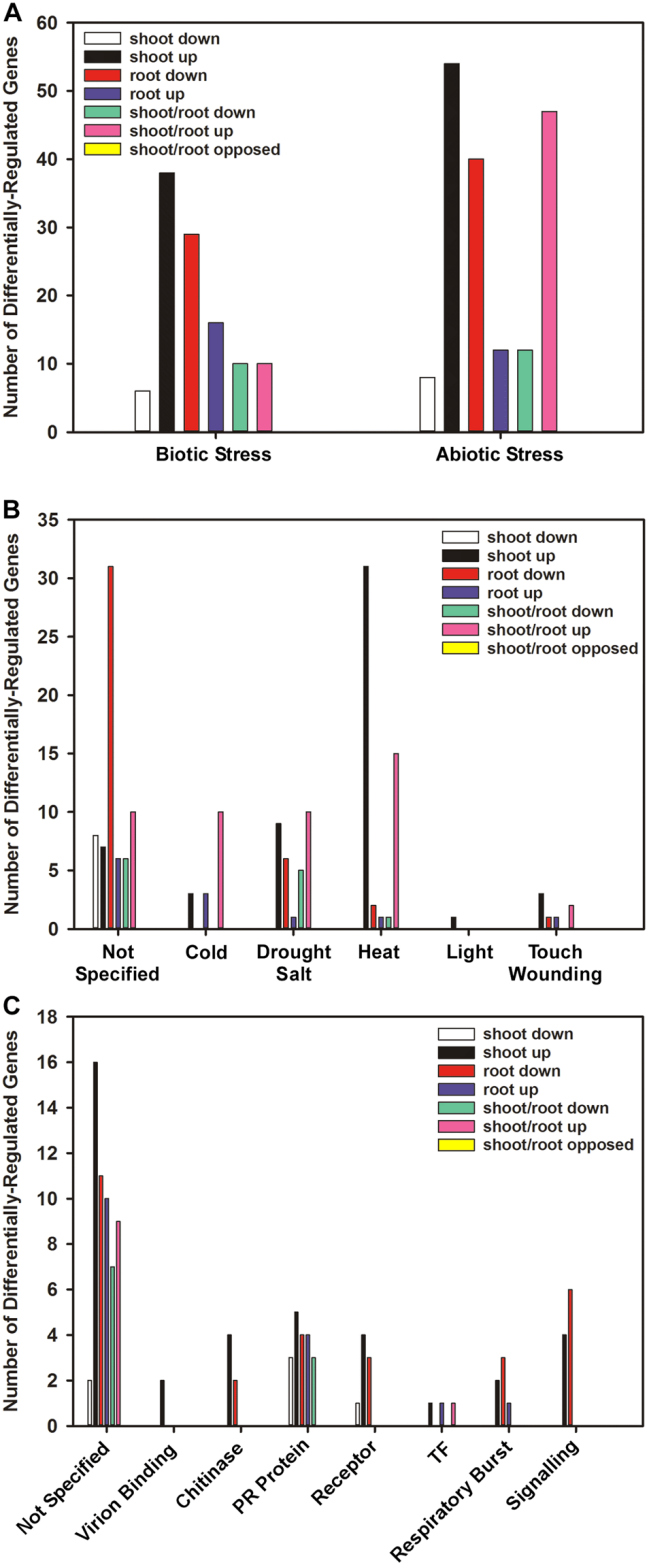
Gene ontology (GO) overview of genes involved in biotic and abiotic stress that were differentially-expressed in *C. sativa* DH55 in response to salt treatment. Panels show a general overview of differentially-expressed genes (**A**) and breakdowns according to the type of stress (**B**) and function (**C**). Genes with an FDR and P value ≤ 0.05 and an absolute value of log 2 FC higher than 1 were considered significant.

The general responses to abiotic and biotic stresses overlap extensively. In salt-treated camelina, stress-responsive genes were generally down-regulated in roots, but up-regulated in shoots (Fig. 9A). More than 50 genes related to abiotic stress were commonly up-regulated in both shoots and roots, while only a few genes related to abiotic stress were commonly down-regulated in both tissues (Fig. 9B). The same trends occurred with genes related to biotic stress, but with just a few genes being commonly up-regulated in shoots and roots (Fig. 9C). The most significantly impacted genes were Csa04g067860 and Csa06g054300 which encode a tryptophan-rich sensory protein/translocator (TSPO) induced by osmotic and salt stress or ABA. These genes were up-regulated 294 and 535-fold in roots and 50 and 131-fold in shoots, respectively. Among the biotic stress-related genes, the expression of those encoding several pathogenesis-related protein (PR) proteins was altered in both roots and shoot tissues (Supplemental Data).

#### Signalling

**Figure 10:**
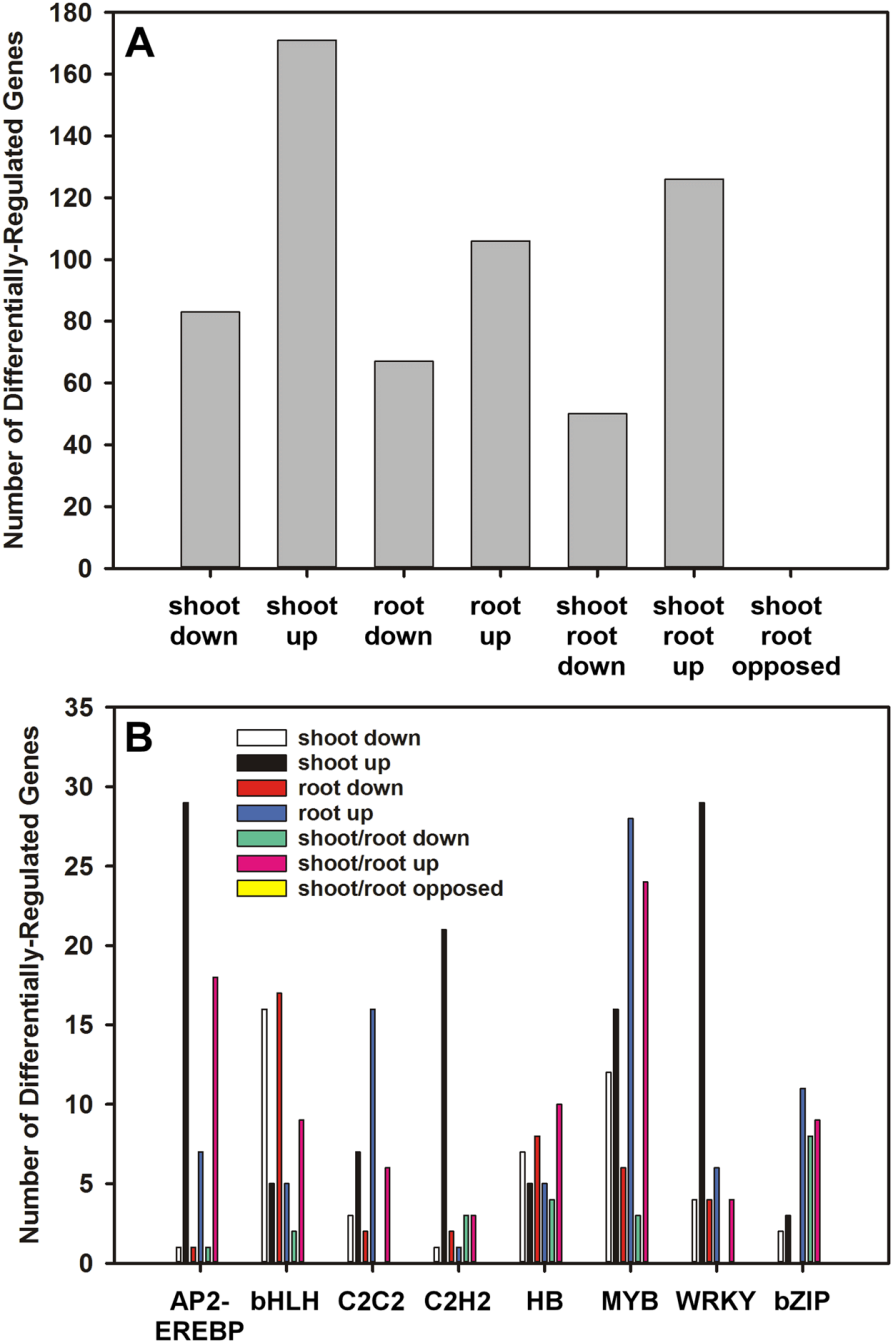
Gene ontology (GO) overview of genes encoding transcription factors that were differentially-expressed in *C. sativa* DH55 in response to salt treatment. Panels show a general overview of differentially-expressed genes (**A**) and a breakdown according to the type of transcription factor (**B**). AP2/Ethylene-Responsive Element Binding Protein family (AP2/EREBP), Basic Helix-Loop-Helix family (bHLH), zinc finger domain-containing protein (C2C2), zinc finger family (C2H2), Homeobox transcription factor family (HB), MYB domain transcription factor family (MYB), WRKY domain transcription factor family (WRKY), bZIP transcription factor family (bZIP). Genes with an FDR and P value ≤ 0.05 and an absolute value of log 2 FC higher than 1 were considered significant.

Coordinating changes in gene expression is an important factor for managing the response to stress and transcription factors (TFs) play a principal role in triggering the correct sequence of responses. In total, 133 genes encoding various TFs were down-regulated in shoots, while 297 were up-regulated in response to salt treatment. In total, 232 genes were up-regulated in roots and only 120 genes were down-regulated. Among these, 53 were commonly down-regulated and 126 were commonly up-regulated in both shoots and roots (Fig. 10A). The MYB TF family represented the largest fraction, 15% of total, of the genes encoding TFs that were differentially-regulated in response to salt treatment in camelina (Fig. 10B; Supplemental Data). Among them, the gene encoding (Csa10g033360) was up-regulated 90-fold in roots, while the other two homeologues (Csa12g063080 and Csa11g041570) were up-regulated in both roots (143 and 191-fold) and shoots (72 and 89-fold). While genes involved in auxin biosynthesis were discussed earlier, several genes encoding auxin/indole-3-acetic acid (Aux/IAA) responsive TFs were down-regulated in shoots and/or roots, for example, those encoding IAA7 (Csa01g027520), IAA13 (Csa16g050050), IAA14 (Csa12g057270), IAA15 (Csa16g050050), IAA17 (Csa17g007430 and Csa14g005400), and IAA34 (Csa17g020620 and Csa03g018850). Csa05g002640 encodes Auxin Response Factor 11 (ARF11) and was down-regulated in both shoots and roots.

**Figure 11:**
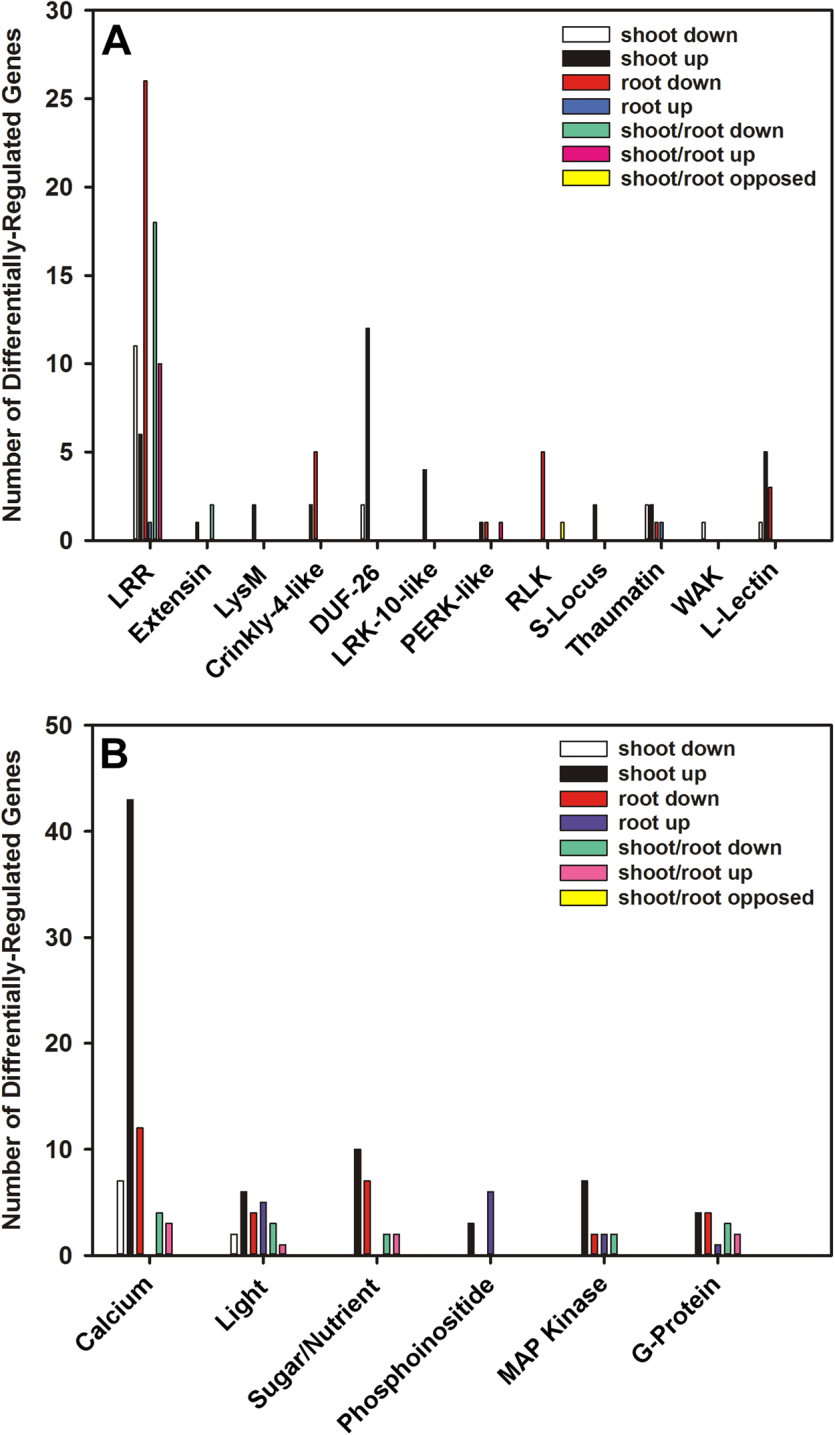
Gene ontology (GO) overview of genes encoding receptor kinases (**A**) and other receptor families (**B**) that were differentially-expressed in *C. sativa* DH55 in response to salt treatment. Genes with an FDR and P value ≤ 0.05 and an absolute value of log 2 FC higher than 1 were considered significant.

Receptors and their cognate signalling pathways have an essential role in plant growth by allowing recognition and response to external stimuli. In total, 17 genes encoding various receptor kinase family proteins were down-regulated and 37 were up-regulated in shoots. In contrast, 41 genes were down-regulated and only 2 genes were up-regulated in roots. 20 genes were commonly down-regulated and 11 genes were commonly up-regulated in shoots and roots (Fig. 11A). Csa12g033490 was the only gene encoding a receptor kinase that was oppositely-regulated in roots compared to shoots. It encodes the Cysteine-rich Receptor-like Protein Kinase (CRK) RLK10 and was down-regulated 163-fold in roots, but up-regulated 47-fold in shoots. In total, 23 genes encoding various other receptor family proteins were down-regulated and 95 were up-regulated in shoots in response to salt treatment, while 43 genes were down-regulated and 36 genes were up-regulated in roots (Fig. 11B).

#### Ion transport

**Figure 12:**
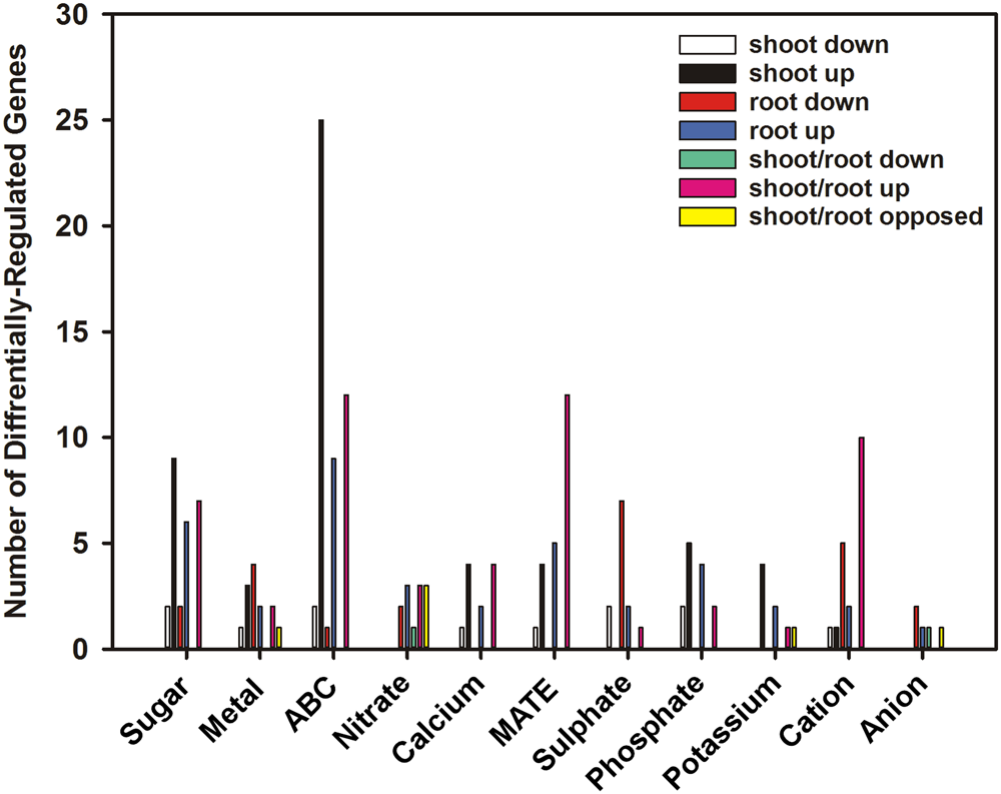
Gene ontology (GO) overview of genes encoding proteins involved in ion or molecule transport that were differentially-expressed in *C. sativa* DH55 in response to salt treatment. Genes with an FDR and P value ≤ 0.05 and an absolute value of log 2 FC higher than 1 were considered significant.

The number of genes encoding proteins with some type of ion transport activity that were differentially-regulated in roots was 2-fold more than the number in shoots (Fig. 12). Genes encoding the K^+^ transporters KT1 (Csa07g013260), KAT1 (Csa11g065760) and HKT1 (Csa13g047450) were up-regulated, as was Csa04g006390 which encodes the Na^+^/H^+^ exchanger. Genes encoding another HKT (Csa08g040240) or the Na^+^/H^+^ exchanger NHX4 (Csa15g007930) were down-regulated in shoots, while Csa08g040240 was up-regulated in roots. Genes encoding K^+^ uptake permease 6 (KUP6) and HKT5 were up-regulated in shoots (Supplemental Data).

In roots, several genes involved in transport of other cations were down-regulated, including those encoding iron-regulated transporter 1 (IRT1) (Csa12g041800, Csa10g024490 and Csa11g027610) (Supplemental Data). A gene encoding a zinc transporter 8 precursor (Csa20g074550) was down-regulated, while that encoding the cadmium-transporting ATPase HMA2 (Csa11g013320) was up-regulated in roots, but down-regulated in shoots. Genes encoding Ca^+2^:cation antiporters (Csa06g026060, Csa13g020680 and Csa20g026040) were up-regulated in both shoots and roots. The other homeologues of Csa06g026060 (Csa09g058900, Csa04g037680) and Csa08g010140 were up-regulated only in shoots, as were genes encoding other Ca^+2^ and cation transporters. Genes encoding a calcium-transporting ATPase (Csa11g090670) and a cation efflux protein (Csa03g020030) were commonly up-regulated in both roots and shoots. In addition to inorganic cations, genes encoding organic cation transporters (Csa09g093580, Csa07g058430, Csa15g031000 and Csa01g023720) were up-regulated in shoots and roots.

Genes involved in anion transport were also highly regulated in shoots and/or roots, including those encoding nitrate transporters NRT1 (Csa17g016300) and NRT2:1 (Csa03g011540 and Csa17g011550) (Supplemental Data). The expression of genes involved in phosphate and sulphate transport were also affected by salt treatment. A gene encoding the sulfate transporter SULTR3;4 (Csa15g020720) was up-regulated in roots and in shoots, while the other two homeologues (Csa01g018620 and Csa19g022820) were up-regulated in roots. In general, genes encoding proteins involved in phosphate transport were down-regulated in roots; however, genes encoding phosphate transporters (Csa16g030840 and Csa05g086190), phosphate transporter 1 (Csa16g040570 and Csa07g047990), and phosphate transporter 4 (Csa08g014220) were up-regulated in shoots. A gene encoding chloride channel B (Csa06g007100) was down-regulated in both roots and shoots, while the other homeologues (Csa04g014930 and Csa09g012970) were down-regulated just in roots. The gene encoding the anion exchanger BOR5 (Csa09g079390) was up-regulated in roots and down-regulated in shoots, while another homeologue encoding BOR5 (Csa16g038700) was up-regulated only in roots.

#### Systems involved in heavy metal management

Plants avoid the toxic effects of heavy metals by either restricting their uptake from soil or by sequestration in ligand-metal complexes^53^. The current study indicated that a broad range of systems for heavy metal avoidance or detoxification were induced in response to salinity stress in camelina (Supplemental Data). All differentially-expressed genes involved in heavy metal exclusion were up-regulated in roots, while some genes encoding heavy metal transporters were up-regulated and others down-regulated in shoots. Csa11g013320 encodes a heavy metal ATPase 2 involved in cadmium and zinc export and was down-regulated in shoots, but up-regulated in roots. Two genes (Csa02g048960 and Csa18g023670) encoding a metal detoxification super family protein were up-regulated in shoots. Csa10g002430 and Csa12g002570 encode heavy metal transport/detoxification superfamily proteins that were up-regulated just in roots, as was a gene (Csa19g035500) encoding Selenium-Binding Protein 3 (SBP3). Three camelina genes (Csa03g026830, Csa14g029230 and Csa17g030050) encoding heavy metal transport/detoxification superfamily protein 8 involved in detoxification of cadmium were up-regulated in roots and shoots. A slight increase in iron concentration was observed in shoots of salt-treated plants (Fig. 4). Indeed, genes encoding the ferric iron binding proteins Ferretin 1 (Csa13g001310, Csa20g001420 and Csa08g063260) were commonly up-regulated in both shoots and roots, while those encoding Ferritin 2 (Csa15g014720 and Csa01g012060) were up-regulated only in roots.

## Discussion

*C. sativa* is considered to be better suited for growth in marginal soils than more widely cultivated oilseed crops; however, its’ inherent tolerance and ability to adapt to abiotic stresses has not been very well-studied. In one study, even moderate salt stress was observed to reduce seed yield considerably^6^. This work examined changes in the biochemistry and gene expression patterns in roots and shoots of camelina plants exposed to salt stress with the aim of identifying mechanisms that could be targeted for further improvement.

### Ion Transport and Metal Detoxification

Soil salinity inhibits macro-element (K, Ca, Mg, P and S) uptake, but also hampers the absorption of micro-elements (Zn, Fe, Mn, Cu, B) leading to nutrient deficiency and metabolic disorders^54^. Treatment of barley with NaCl results in a significant reduction in Ca and Mg content in seedlings^55^ which is in contrast to our study in camelina where Mg concentration increased and Ca was not affected. Salt composition is also a contributing factor in salt tolerance. In the current study, the effect of NaCl on camelina growth and development was more severe than that observed in a previous study which used a saline solution composed of four salts (CaCl_2_, NaCl, MgSO4.7H_2_0, Na_2_SO4) resembling the composition found in soils on the Canadian prairies^3^. Plants treated with NaCl at EC 20 dSm^−1^ died after 35 days, whereas plants treated with the saline solution at the same EC survived and produced seed^3^. Camelina was tolerant to both NaCl and the natural saline solution at EC 15dSm^−1^.

Influx of Na^+^ through ion channels or other membrane transport systems disrupts the EC inside the root. To counteract this, cation:proton anti-porters (CHX) efflux Na^+^ into the soil or the cortical apoplast, while high affinity potassium transporters (HKT) import K^+^ into root cells^56^. In accordance, genes encoding K^+^ transport channel proteins were up-regulated in roots, including Csa11g065760 (KAT1). Rice OsKAT1 participates in the maintenance of cytosolic cation homeostasis during salt stress^57^. Csa04g006390 encodes the Na^+^/H^+^ transporter (HKT) orthologous to AtSOS1 and was also up-regulated. SOS1 exports Na^+^ to the extracellular space and imports H^+^ into the cell. Over-expression of *SOS1* greatly improves salt tolerance in transgenic plants^58^. In camelina shoots, the gene encoding the Na^+^/H^+^ exchanger NHX4 (Csa15g007930) was down-regulated. In Arabidopsis, *NHX4* disruption increased tolerance to salt stress^59^. Csa15g006210 encodes the vacuolar Na^+^, K^+^/H^+^ antiporter NHX2 which was up-regulated in roots and shoots. In Arabidopsis, AtNHX2 is crucial for K^+^ uptake by the tonoplast to regulate stomatal opening and closure during drought and salt exposure^60,61^. The high demand for K^+^ in aerial tissues is met through uptake of K^+^ from the soil and further translocation to the aerial parts of the plant. Camelina genes encoding K^+^ uptake permease 6 (KUP6) and HKT5 were up-regulated in shoots. KUP, TRK and HAK-type transporters are responsible for the bulk of K^+^ accumulation in plants and expression of Arabidopsis *AtKUP1* increases K^+^ uptake^62^.

The concentration of heavy metals (Cd, Pb and Ni) increases in soil concomitant with increasing salinity resulting in an increase in absorbed heavy metals in all parts of the plant^63^. Numerous genes encoding proteins involved in heavy metal transport/detoxification were up-regulated in camelina roots in response to salt, including a gene (Csa19g035500) encoding Selenium-Binding Protein 3 (SBP3). In Arabidopsis, SBP1 expression increases Cd tolerance and *in vitro* experiments showed that SBP1 binds Cd^64^. Taken together, the gene expression data indicates that in response to salt stress in camelina, ion movement is directed toward the efflux of Na^+^ and increasing K^+^ and Ca^+2^ concentration in the tonoplast and cytoplasm. In Arabidopsis, mutants with increased salt tolerance also exhibited higher rates of Cl^−^ and Na^+^ exclusion and absorption of K^+^^65^. Increasing cytosolic Ca^+2^ is also important for activating the SOS pathway which is involved in maintenance of ion homeostasis^65^. Other studies have shown that phosphate and sulphate transporters work to assure supply during exposure to stress conditions^66,67^ and the gene expression data supports this notion. In addition, the current study indicates that camelina uses a wide variety of mechanisms to avoid heavy metal toxicity, including exclusion and chelation, and that heavy metal handling is an important component of the salt stress response in camelina.

### Metabolism

In this and other studies, salt treatment was shown to negatively impact photosynthetic ability which reduces energy conversion of absorbed light by chloroplasts with excess light energy leading to photo-inhibition. Photo-inhibition reduces the quantum yield of PSII and induces photorespiration and H_2_O production^68^. In camelina, many genes related to PSI, PSII and the Calvin cycle were down-regulated in response to salt, while genes involved in photorespiration were up-regulated. Enhancement of photorespiration confers resistance to salt in rice^69^ and this may be an avenue to further improve salt tolerance in camelina. Interestingly, though not a photosynthetic tissue, photosynthetic genes are differentially-regulated in roots under stress^70^. In camelina, a gene related to PSII (Csa06g008820) was up-regulated around 200-fold in salt-treated roots, but its function here is unclear.

A shift in carbohydrate metabolism leading to the accumulation of soluble sugars and osmolytes is a classic response to maintain cell turgor and to protect cell membranes from damage under salt and cold stress^41,71^. In camelina, several genes encoding amylases were differentially-regulated. β-amylase plays a major role in transitory starch breakdown into maltose^72^ and levels of this enzyme increase in response to osmotic, salt, cold and heat stress^71,73^. It was suggested that maltose could function as a compatible solute during stress as it is known to protect proteins from degradation, stabilize plant membranes and contribute to osmotic potential^71^. Synthesis and accumulation of sugar alcohols, such as mannitol, sorbitol, and sucrose, with osmo-protectant properties is a common feature of salt-tolerant species^74^, and several genes involved in the synthesis of sucrose and other minor carbohydrates (e.g. inositol, raffinose, trehalose) were up-regulated in camelina in the presence of salt. Sucrose and glucose act as substrates for cellular respiration and as osmolytes to maintain cell homeostasis^75^, while fructose is involved in the synthesis of secondary metabolites, lignin and phenolic compounds^76^. Sugars accumulated during drought stress can also be used as an energy source for rapid recovery once water becomes available^77^. The ability to scavenge water goes hand-in-hand with balancing osmotic potential. Some of the most highly up-regulated genes encoded a tryptophan-rich sensory protein/translocator (TSPO). TSPO is involved in regulating the cell-surface expression of plasma membrane aquaporins during abiotic stress, specifically salt stress^78^.

Based on gene expression patterns, respiration (glycolysis, fermentation, mitochondrial function) appeared to be profoundly affected in camelina under salt stress. Salt stress increases the amount of the hexose-P in the cytosol which can be used as an osmolyte or as an energy resource for the plant during stress conditions. In addition, hexose-P can enter the triphosphoinositol pathway and serve as secondary messenger in stress signal transduction, probably activating the SOS signaling pathway^79^.

**Figure 13:**
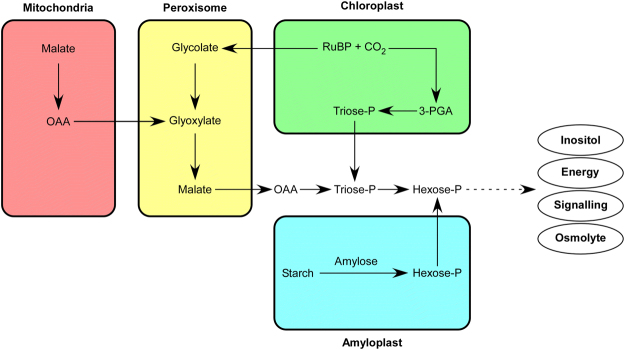
Model depicting carbohydrate partitioning and flux within organelles in camelina in response to salt treatment as predicted from gene expression patterns. OAA: oxaloacetate, 3-PGA: 3-phosphoglycerate and RuBP: ribulose 1,5-biphosphate.

Saline, osmotic and drought stresses lead to reduced availability of NADP^+^, as well as an increase in photosynthetic electron leakage and reactive oxygen species (ROS). Aldehyde dehydrogenase is responsible for recycling NAD or NADP during anoxia caused by salt stress and several genes encoding these enzymes were up-regulated in camelina shoots under salt stress. Salt stress also disrupts mitochondrial function leading to decreased electron transport activity, increased mitochondrial ROS generation and lipid peroxidation. Mitochondrial alternative oxidases, of which several were up-regulated in camelina, increase salt tolerance by sustaining metabolic and signaling homeostasis in response to salt stress^80^. Fermentation underpins cytoplasmic pH regulation and prevents ROS production by reducing the time that electrons are sequestered in the photosynthetic electron transport chain^81^ and gene expression patterns in camelina indicate a shift toward fermentation under salt stress. Based on the changes in the expression of genes involved in photosynthesis, fermentation, minor and major carbon metabolism, and Krebs cycle, a model can be developed to predict shifts in major metabolites in shoots and roots of salt-stressed camelina plants (Fig. 13).

Increased nitrogen metabolism is observed in halotolerant plants, such as *Salicornia europaea*^42^, and in roots of salt-resistant rice cultivars^43^. Over-expression of glutamine synthetase enhances salt tolerance in Arabidopsis^44^, and the expression of glutamine synthetase 2 is related to enhancement of photorespiration and resistance to salt in rice^69^. In contrast, genes encoding glutamine biosynthetic enzymes were down-regulated in camelina in response to salt. Similarly, increasing NaCl concentration decreased nitrate uptake in tomato seedlings due to a reduction in the expression of genes encoding nitrate transporters and glutamine synthetases^82^. In camelina, as in tomato, the relationship between the expression of genes involved in nitrogen uptake and assimilation appears incongruent with that observed in halophytic species. Furthermore, in addition to its role in nitrogen assimilation, glutamine synthetase is also involved in the *de novo* synthesis of the osmo-protectant amino acid proline^83^ and an overall increase in amino acid content is observed during drought and salinity stress^41^. In particular, the gene encoding pyrroline-5-carboxylate synthase (P5CS) was up-regulated in camelina shoots. This is a key enzyme in the synthesis of pyrroline-5-carboxylate, an intermediate in the synthesis of proline and ornithine and these amino acids contribute to redox balance, osmotic and salt stress tolerance in plant cells^84^. Taken together, it appears that camelina may employ a slightly different mechanism in responding to salinity stress than that observed in some other plants.

### Growth and development

Changes in cell wall structure is a common feature of the plant response to salt stress^85^ and is reported to be an acclimation strategy in salinity stress in glycophytic plants^86^. Maintaining cell wall integrity and cell turgor pressure by increasing cellulose synthesis permits constant cell growth under low water potential^87^. In maize, sustained salt stress impairs epidermal wall-loosening in leaves due to limited abundance of cell wall-loosening β-expansin proteins; however, salt-tolerant cultivars have an increased amount of these proteins and are able to maintain leaf growth under saline conditions as the epidermal cell wall is more extensible^88^. The current study demonstrated that the expression of cell wall-related genes, including those encoding several expansins, was affected in camelina in response to salt. Interestingly, genes encoding pectin methyl-esterase inhibitor (PMEI) were down-regulated in roots, but up-regulated in shoots during salt treatment. Pectin methyl-esterase (PME) activity is regulated by PMEI and plays a key role in osmotic stress by increasing cell wall fortification^89^.

Changes in growth and development that result from, and are an adaptation to, salt stress is also governed by hormones. In camelina under salt stress, genes involved in the auxin biosynthesis appear to be up-regulated in shoots, while genes involved in auxin transport/distribution (PIN2, EIN1) were down-regulated in roots. Auxin is transported downward from the stem and stimulates root development, but high auxin concentrations inhibit root elongation. It should be noted that in camelina root length was not affected by salt stress, whereas root mass was. Higher auxin levels resulting from over-expression of the auxin biosynthesis-related gene, *YUCCA3*, increases hyper-sensitivity to salt in Arabidopsis^90^. Furthermore, maintenance of meristem in roots is the result of changes to the distribution of auxin transporters (AUX1, PIN1/2) which become more diffusely distributed during salt stress^28^. GA negatively affects salt tolerance and mutants deficient in GA biosynthesis or signaling display enhanced survival under severe salt stress^47^. In camelina, many genes involved in GA synthesis and signaling were down-regulated in response to salt treatment suggesting that GA levels fall dramatically as they do in other plants^91^. Conversely, BR has a positive impact on salt and drought stress^48,92^, and higher BR levels decrease water loss under such conditions by reducing stomatal conductance and density under salt stress^93^. However, many of the genes involved in BR synthesis and signalling were down-regulated in camelina roots and shoots in response to salt, including BR Suppressor 1 (BRS1) (Csa10g011910 and Csa12g016550), a protein involved in an early event in BR signaling^94^. Ethylene levels profoundly affect salinity stress as low levels induce stress tolerance, while high levels negatively impact plant growth^3,50,51^. Many genes involved in ethylene biosynthesis and signaling were up-regulated in camelina shoots in response to salt, including several encoding ERFs. Over-expression of ERF genes has been shown to enhance salt tolerance in tomato^95^, while an Arabidopsis ethylene-insensitive mutant (*etr1-1*) exhibited increased sensitivity to salt^96^. Several genes encoding enzymes involved in JA biosynthesis were up-regulated in shoots and/or roots in camelina. In rice, the concentration of JA increases during salt stress and higher JA accumulation was observed in salt-tolerant cultivars. It was suggested that JA protects cells from the adverse effects of salt by reducing Na^+^ accumulation^46^. Reversal of damage caused by salt on seedlings and improved photosynthetic activity were also observed after JA application in several crops^49^. ABA helps plants acclimate to low water conditions by closing stomata and promoting the accumulation of osmo-protectants and many genes related to the ABA pathway were up-regulated in camelina. However, as with ethylene, the levels and timing of delivery of ABA must be finely regulated as attempts to increase salt tolerance via application of exogenous ABA results in developmental defects^97–99^. Since there is tight crosstalk between the different hormone pathways, it is very difficult to arrive at a conclusion as to what role individual hormones play in root and shoot salt stress in camelina. Based on this gene expression study, ABA signalling appears to be more highly regulated in roots than in shoots in response to salt stress. BR and SA are mostly negatively regulated, while ethylene and JA are positively regulated in both tissues. GA seems to be positively regulated in roots and probably negatively regulated in shoots. Cytokinin is not affected in roots and a balance between negative and positive regulators was observed in shoots.

### Regulation of gene expression

In Arabidopsis, drought stress increases the expression of about 51% of the entire genome allotment of MYB TF genes and decreases the expression of 41%^100^. In camelina, the largest group of TF genes induced in response to salt stress were those encoding MYB factors. Some MYB TFs positively regulate gene expression, while others negatively regulate the response of the plant to stress stimuli. For example, Arabidopsis *AtMYB2*, *AtMYB74*, and *AtMYB102* showed enhanced expression under drought stress^101^ and the camelina *MYB2*, *MYB102* and *MYB15* orthologues were also induced in shoots under salt stress. AtMYB15 is involved in ABA-, ethylene-, and JA-mediated signalling in response to salt, cold and water deprivation, and lines over-expressing *AtMYB15* display improved drought and salt stress tolerance^102^. AtMYB20 is involved in ABA signaling in response to salt and desiccation stresses^100^ and the camelina gene encoding MYB20 was induced in roots in response to salt, as were those encoding MYB108 and MYB121. Arabidopsis lines over-expressing *AtMYB20* exhibit enhanced salt tolerance. AtMYB108 plays a role in the response to abiotic stresses (salt, drought, and cold) that are regulated by JA through ROS-mediated signaling^103^ and is closely related to *AtMYB2*, which is involved in the crosstalk between abiotic and biotic stress pathways^101,103^. MYB121 is involved in the induction of salt, drought and cold stress responses in tomato. Tomato plants over-expressing apple *MYB121* accumulated less Na^+^, exhibited a lower Na^+^/K^+^ ratio, had lower levels of electrolyte leakage and a higher proline content under salt stress^104^.

Csa12g033490 was the only gene encoding a receptor kinase that was oppositely-regulated in roots compared to shoots. This gene encodes the Cysteine-rich Receptor-like Protein Kinase (CRK) RLK10 and was down-regulated 163-fold in roots, but up-regulated 47-fold in shoots. CRKs constitute a large subfamily of receptor-like proteins. Mutation of *CRK5* in Arabidopsis impairs stomatal conductance, accelerates senescence, leads to ROS accumulation, and increases foliar levels of ethylene and salicylic acid^105^.

## Conclusion

This study examined changes in the biochemistry and gene expression patterns in roots and shoots of camelina plants exposed to salt stress. Element analysis revealed no significant differences in micro-element concentration in shoots of salt-treated plants. A small decrease in macro-element concentration (P and S) was observed; however, the concentration of Ca and Mg increased significantly after salt treatment. This may indicate adaptation to maintain osmotic homeostasis by increasing the concentration of specific macro-elements involved in plant signaling and photosynthetic function. Maintaining high levels of important plant signaling components, such as Ca, and those required for coenzymes, such as Mg, Fe, and B, may also be part of the tolerance mechanism in camelina to keep critical systems functional during salt stress.

In camelina shoots, genes encoding enzymes involved in the synthesis of secondary metabolites including lignin, anthocyanin and wax were up-regulated; this may imply that mechanisms to improve desiccation resistance and water-use efficiency during salt stress were also induced. In other plants, a hydrophobic wax layer covers the aerial epidermis of terrestrial plants and provides protection against desiccation and environmental stresses^106^, while anthocyanins are photo-protective^107^.

Redistribution of Na^+^ from the cytoplasm to the tonoplast and increasing K^+^ and Ca^++^ concentration of the cytoplasm are other mechanisms to reduce ion toxicity during the salt stress. In camelina roots, gene expression profiles indicated that Na^+^ was exported from the cytoplasm by the SOS pathway and that specific transporters transferred K^+^ from soil to the cytoplasm of root cells.

The up-regulation of genes involved in metal handling, chelation and storage was highly apparent in shoots of salt-treated camelina plants, but not in roots. In shoots, detoxification of heavy metals likely occurred via compartmentalization, at least for Cd and probably Zn, possibly within vacuoles. However, camelina likely employs some type of export mechanism to exclude heavy metals from roots.

Reduction in the expression of genes involved in nitrate uptake and nitrogen assimilation may be one of the reasons for the decreased growth of salt-insensitive plants during severe salt stress; this was also observed in salt-stressed camelina. Armed with this information, breeders may wish to focus on genes which improve nitrate uptake and nitrogen assimilation as an indirect means to increase growth and productivity of camelina in saline soils.

Partial genome partitioning was observed in roots and shoots based on the expression of homeologous genes from the three *C. sativa* sub-genomes. Some members of homeologous groups were expressed in only one tissue, while others were expressed at comparatively different levels in both roots and shoots. We did not, however, observe a situation where homeologues were oppositely-regulated in shoots and roots. The involvement of sub-genome I and II in response to salinity stress seems to be of about the same degree, while about 10% more differentially-expressed genes were associated with sub-genome III.

In conclusion, this study has provided valuable information and insight into the response of camelina to salt stress. This is particularly important as the crop is being promoted as being suitable for growing on marginal lands. Examination of this data and comparison to similar studies in more halophytic species will contribute to the development of even more salt-tolerant varieties of this emerging industrial crop.

## Electronic supplementary material

Supplementary Information

Supplementary Dataset 1
