# Supplementary material for: Changes in gene expression in Camelina sativa roots and vegetative tissues in response to salinity stress
Source: Sci Rep. 2018 Jun 28;8:9804. doi: 10.1038/s41598-018-28204-4 (PMC6023900; doi:10.1038/s41598-018-28204-4)
Supplement: Supplementary file 1 — Supplementary Information [file 41598_2018_28204_MOESM1_ESM.pdf]

Title Page:

Changes in gene expression in *Camelina sativa* roots and vegetative tissues in response to salinity stress.

Author List:

Zohreh Heydarian,

Min Yu,

Margaret Gruber,

Cathy Coutu,

Stephen J. Robinson,

Dwayne D. Hegedus

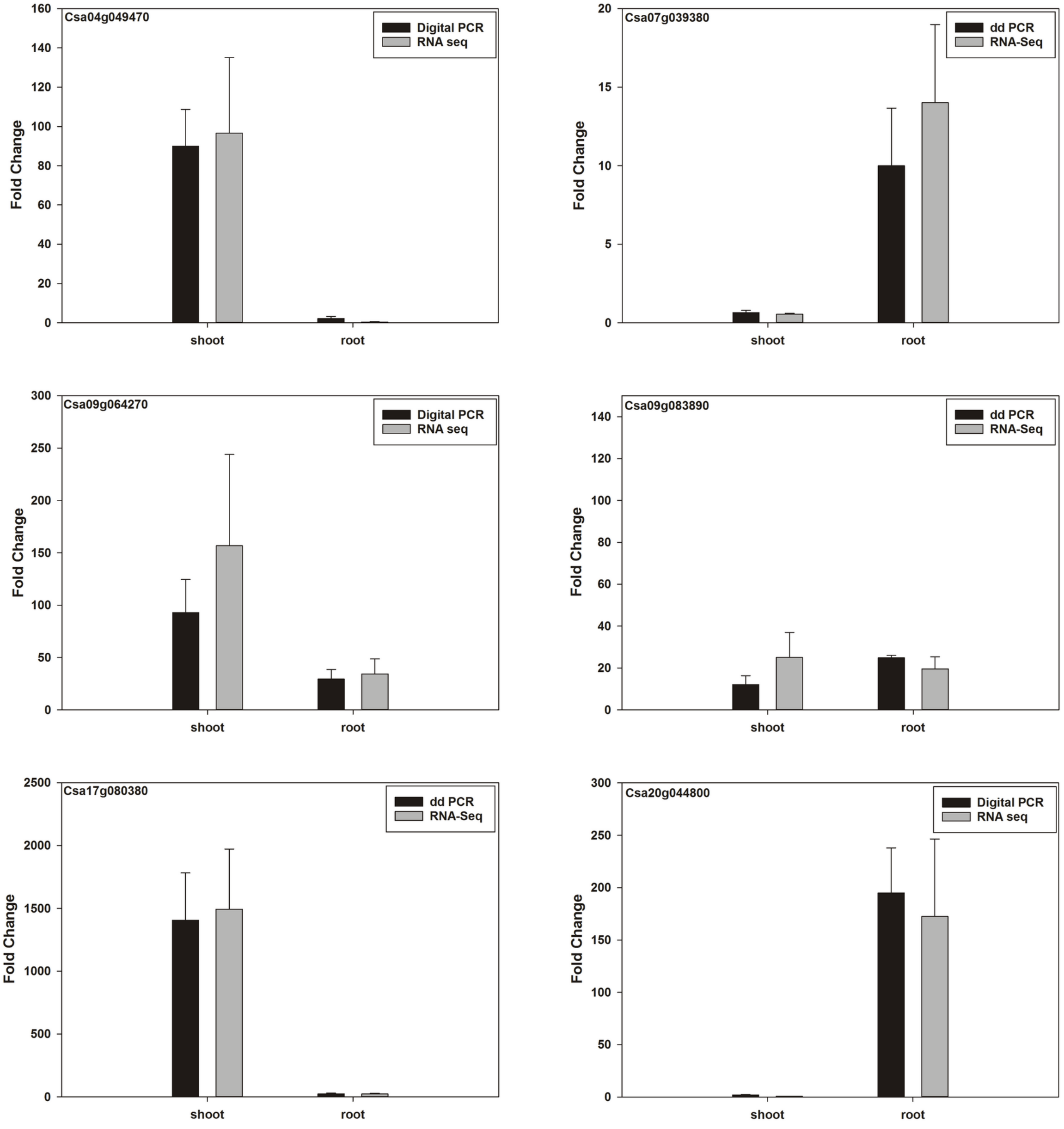

Supplementary Figure 1. Expression of select *C. sativa* genes after salt stress (15 dSm-1) as determined by RNA-Seq and droplet digital PCR (dd PCR) analysis. Plots show expression (fold change) relative to untreated control and are reported as means and standard errors of three biological replicates.

Title: Changes in gene expression in *Camelina sativa* roots and vegetative tissues in response to salinity stress.

Author List:
Zohreh Heydarian,
Min Yu,
Margaret Gruber,
Cathy Coutu,
Stephen J. Robinson,
Dwayne D. Hegedus

Supplemental Figure 2. MAPMAN Pathways

Shoots (Cell Function Response)

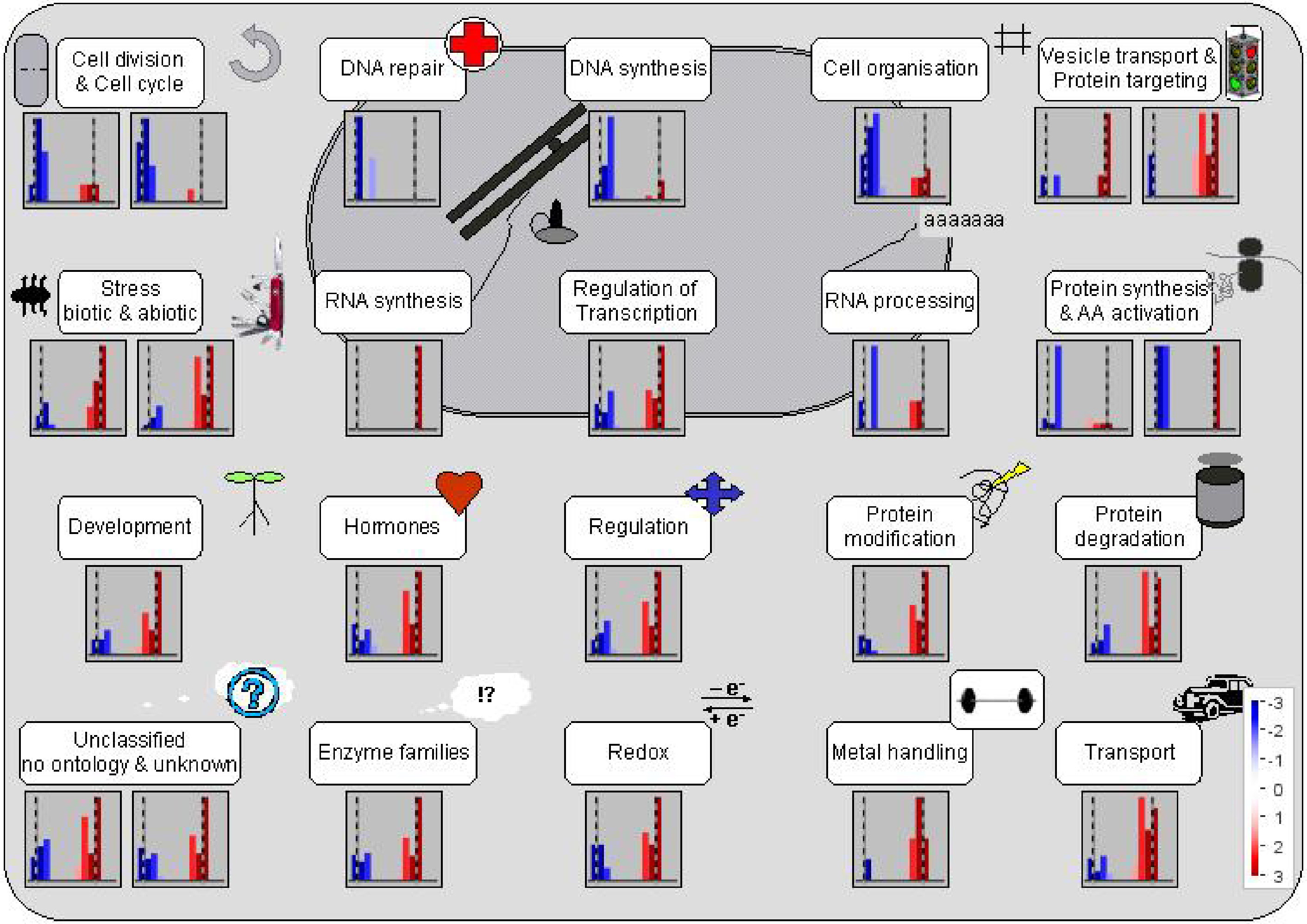

Shoots (Cellular Response)

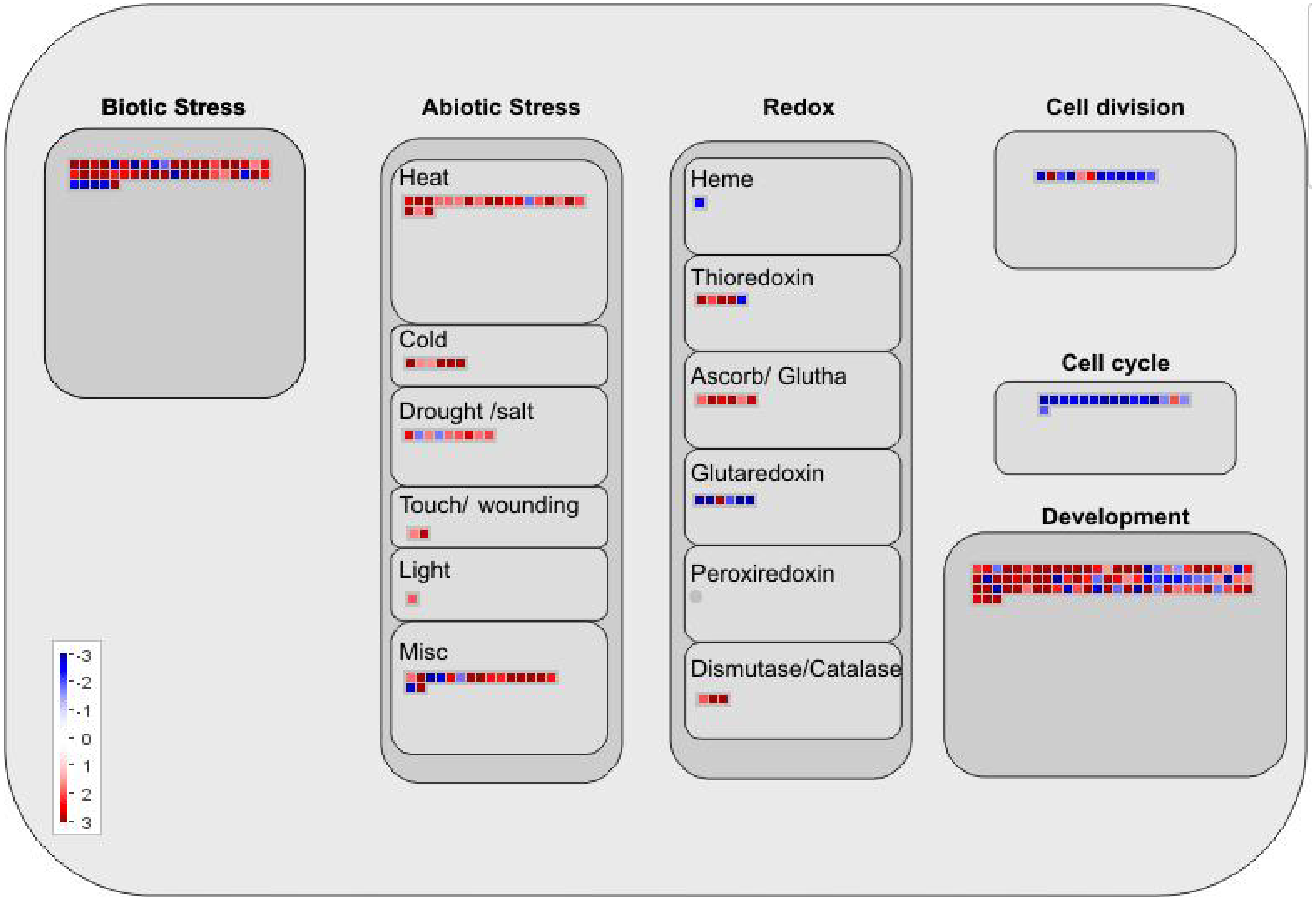

Shoots (Metabolism)

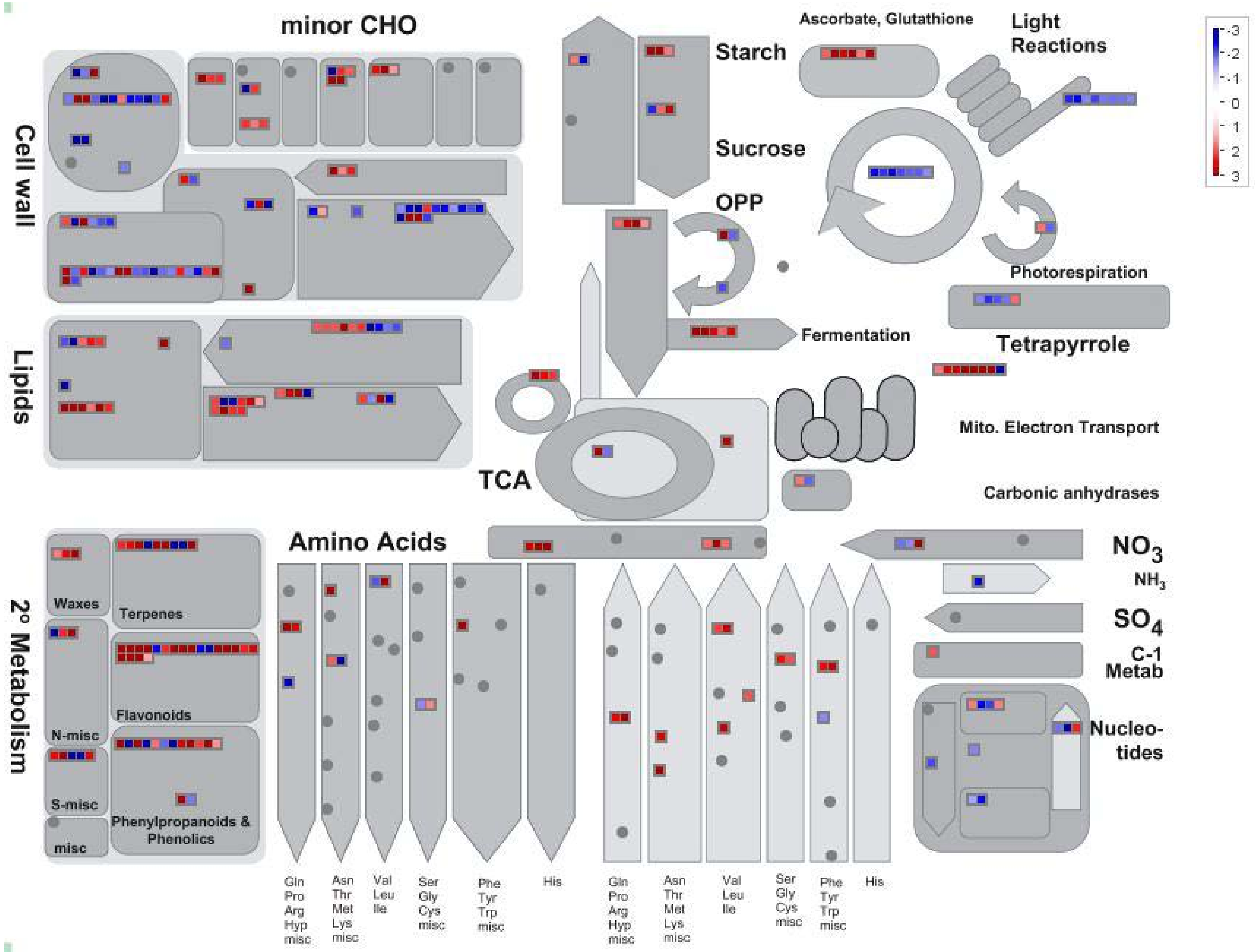

Shoots (Regulation)

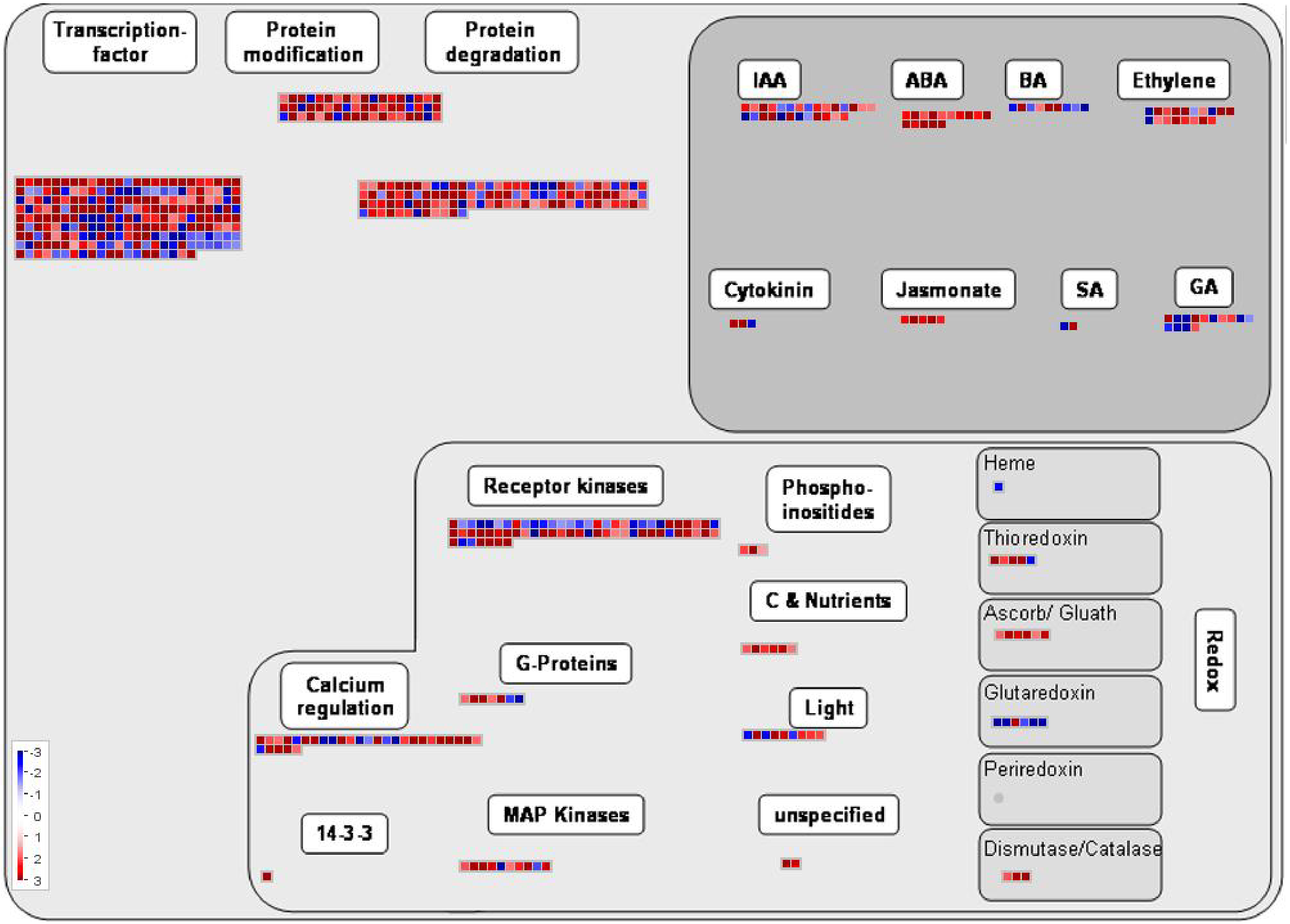

Shoots (Transcription Factor)

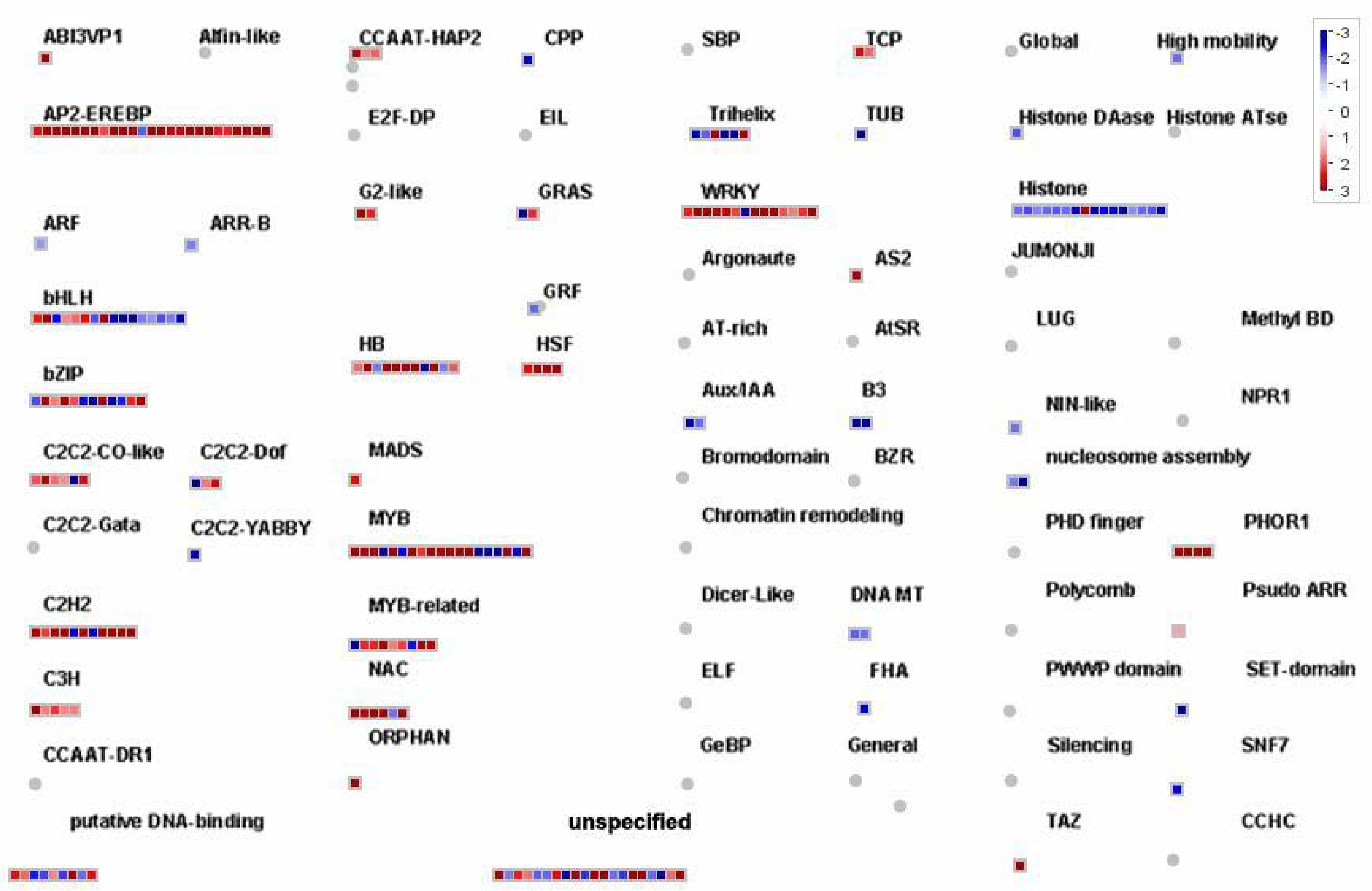

Roots (Cell Function Response)

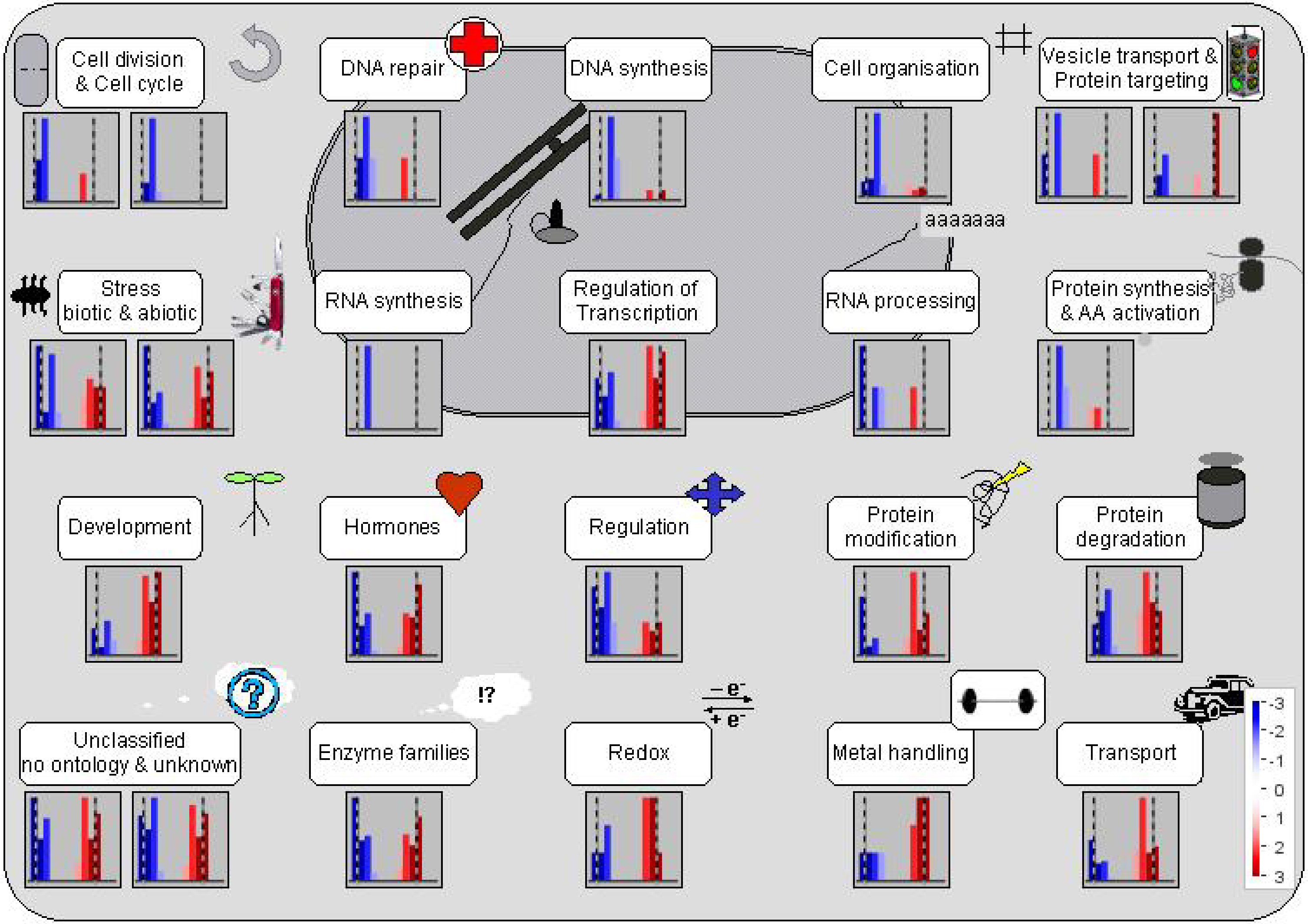

Roots (Cellular Response)

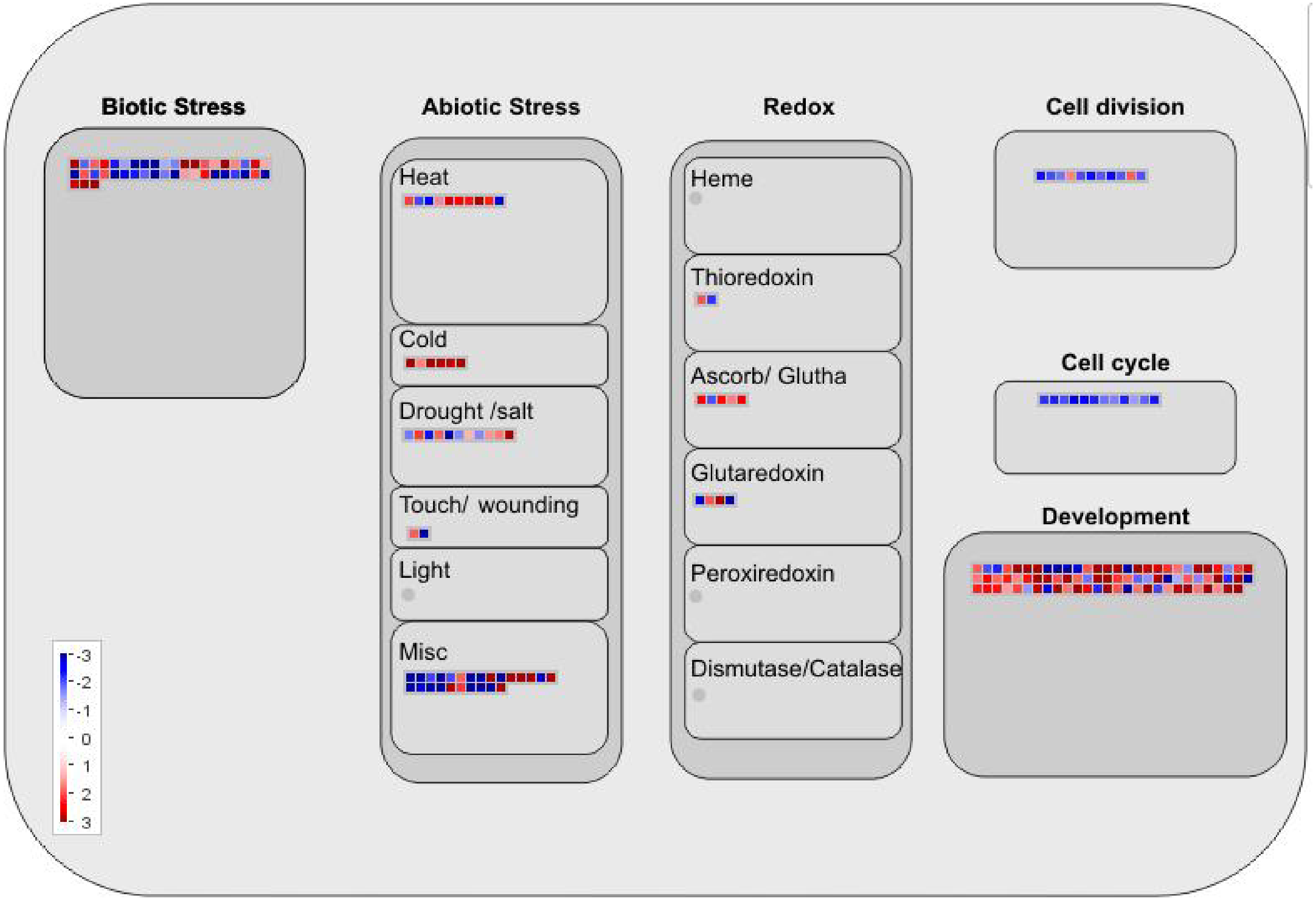

Roots (Metabolism)

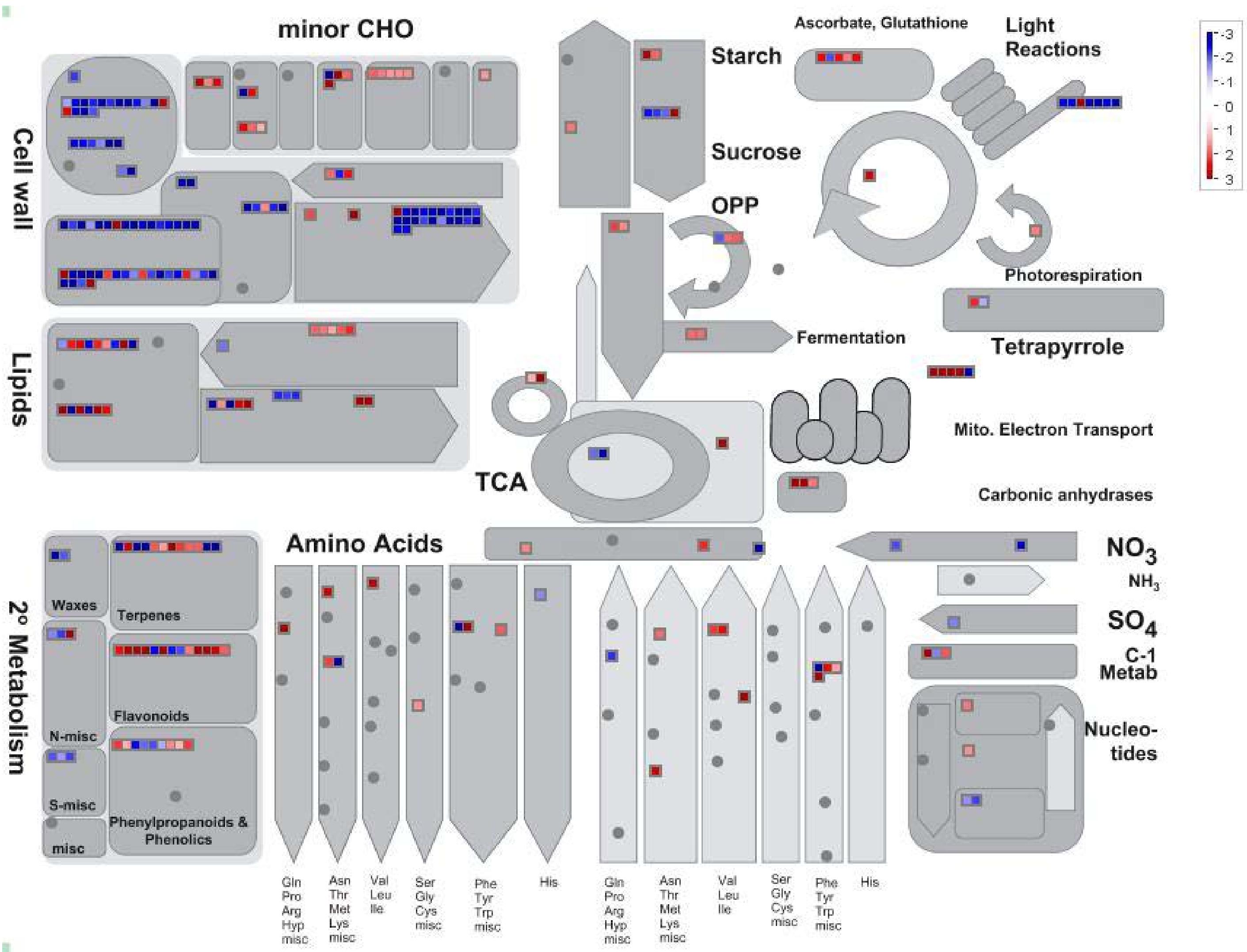

Roots (Regulation)

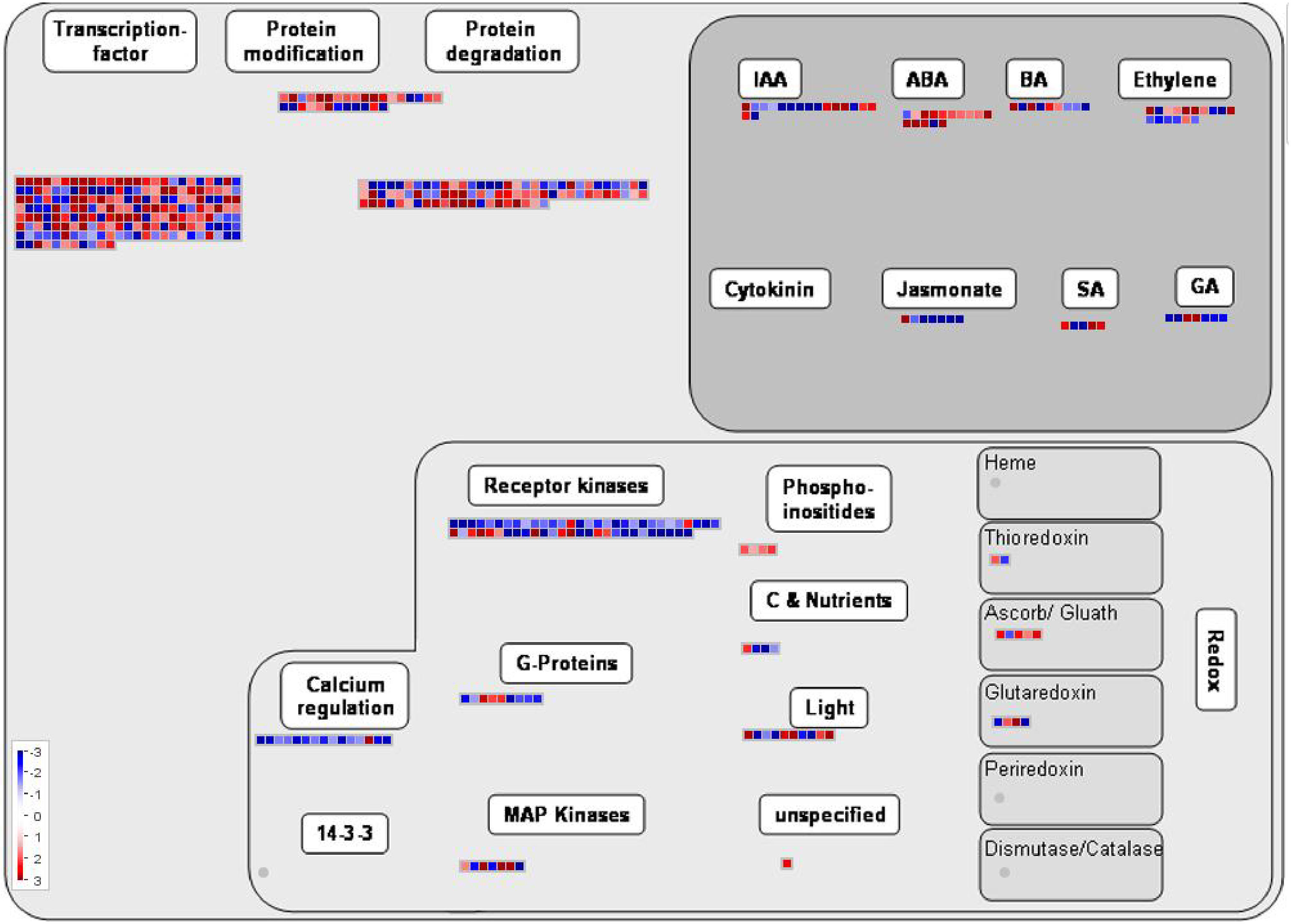

Roots (Transcription Factor)

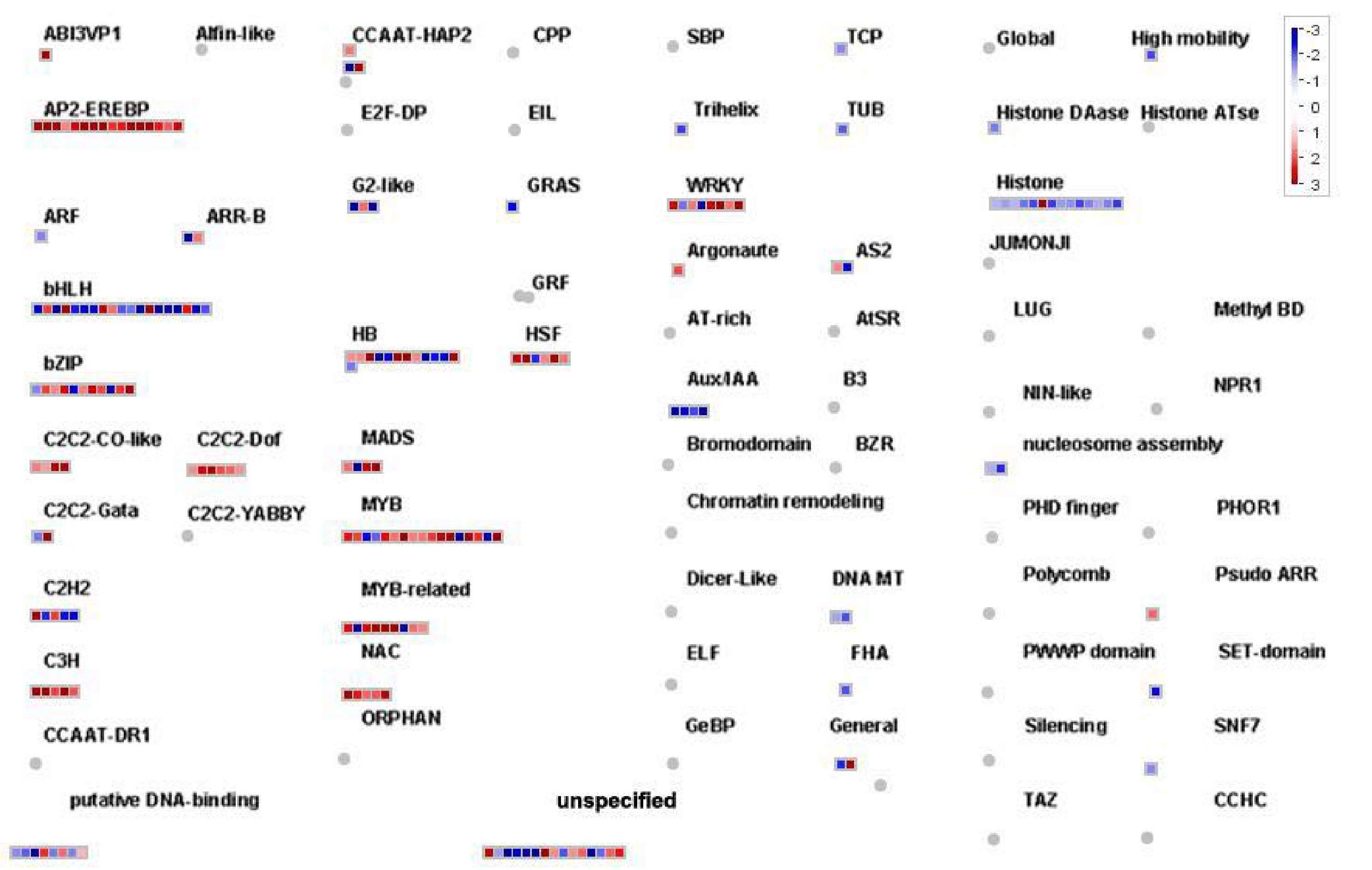

Title Page:

Changes in gene expression in *Camelina sativa* roots and vegetative tissues in response to salinity stress.

Author List:
Zohreh Heydarian,
Min Yu,
Margaret Gruber,
Cathy Coutu,
Stephen J. Robinson,
Dwayne D. Hegedus

Supplemental Table 1. Primers and Probes used for dd PCR analysis

Name	Sequence
CSA04G049470 Forward	ATGGAGCGGAACGTAAACGTGAAC
CSA04G049470 Reverse	TCTGCTCGTTTGCTTCTCTAGCGATC
CSA04G049470 Probe	CTCCAAGACCGCTGAACAAATCTCTG
Csa07g039380 Forward	CCGCCATTACATTTCCCTTCTGT
Csa07g039380 Reverse	CCTCTGTTTATATGAGAAGCTAACCGG
Csa07g039380 Probe	CCCAAACACTCCTTCCGTACCAG
Csa09g064270 Forward	GCAAAACAGTACATGATCTGCTGAGGA
Csa09g064270 Reverse	ATAGATGTTTTCAAACTGTCCTGACTTGTTGG
Csa09g064270 Probe	AGGGAGAAAGCTATTATTGCCAATGGTG
Csa09g083890 Forward	GTGACTATGGCATAAACAGCCCACTAGA
Csa09g083890 Reverse	GCTGGTTGTGTCACATGAGCAGG
Csa09g083890 Probe	TGATGATTCAGAATCAGAGGCTGAGAC
Csa17G80380 Forward	CCGGCCATCAAAATGTAATTCAGAG
Csa17G80380 Reverse	CGTTAACAACCACAACCTTGTCTCTC
Csa17G80380 Probe	TGCCCCGACATCAACCAAAGT
Csa20g044800 Forward	GAATGCATCATATCTGTTGGTGGAAG
Csa20g044800 Reverse	GCGTCCATGGAATCCAAGAAGCTTTCCTC
Csa20g044800 Probe	CAGTTCTGATCAAATCGTTGTTCAAAC
Camelina actin 2 Forward	GCTCTTCCATCGAGAAGAACTAC
Camelina actin 2 Reverse	CAAACGAGGGCTGGAATAAGA
Camelina actin 2 Probe	TGGGCATCTGAATCTCTCAGCACC
